# Expression of an extremophilic xylanase in *Nicotiana benthamiana* and its use for the production of prebiotic xylooligosaccharides

**DOI:** 10.1038/s41598-022-19774-5

**Published:** 2022-09-21

**Authors:** David Talens-Perales, María Nicolau-Sanus, Julio Polaina, José-Antonio Daròs

**Affiliations:** 1 Department of Food Biotechnology, Institute of Agrochemistry and Food Technology, Spanish National Research Council (IATA-CSIC), Paterna, Valencia Spain; 2 Instituto de Biología Molecular y Celular de Plantas (Consejo Superior de Investigaciones Científicas-Universitat Politècnica de València), 46022 Valencia, Spain

**Keywords:** Biochemistry, Biotechnology, Microbiology, Plant sciences

## Abstract

A gene construct encoding a xylanase, which is active in extreme conditions of temperature and alkaline pH (90 °C, pH 10.5), has been transitorily expressed with high efficiency in *Nicotiana benthamiana* using a viral vector*.* The enzyme, targeted to the apoplast, accumulates in large amounts in plant tissues in as little as 7 days after inoculation, without detrimental effects on plant growth. The properties of the protein produced by the plant, in terms of resistance to temperature, pH, and enzymatic activity, are equivalent to those observed when *Escherichia coli* is used as a host. Purification of the plant-produced recombinant xylanase is facilitated by exporting the protein to the apoplastic space. The production of this xylanase by *N. benthamiana*, which avoids the hindrances derived from the use of *E. coli*, namely, intracellular production requiring subsequent purification, represents an important step for potential applications in the food industry in which more sustainable and green products are continuously demanded. As an example, the use of the enzyme producing prebiotic xylooligosdaccharides from xylan is here reported.

Plants have emerged as a promising alternative for heterologous protein production^1^. Plant growth is fueled by sunlight, and production can be scaled up quickly and easily. As in other eukaryotic organisms, in plants, proteins are subjected to post-translational modifications and can be targeted to the export pathway or accumulated in specific subcellular localizations. Additionally, if production is properly managed, plant products are free of human pathogens, which is particularly important in the pharmaceutical and cosmetic sectors^2,3^. Plants can be stably transformed to program production of recombinant proteins. However, this is a time-consuming process involving, in most species, labor-intensive regeneration steps based on tissue culture techniques^4^. In addition, a high production yield is not always achieved. As an alternative, transient expression strategies that use the ability of *Agrobacterium tumefaciens* to efficiently transfer a DNA fragment (T-DNA) to plant cells have been developed^5^. These strategies, which are often based on regulatory elements derived from plant viruses, are technically simpler and frequently achieve higher yields^6^. Vectors based on tobacco mosaic virus (TMV; genus *Tobamovirus*, family *Virgaviridae*) have been used to produce recombinant proteins in plants, owing to the intrinsic capacity of this virus to produce enormous amounts of the capsid protein (CP) in plant cells. This capacity is likely based on the efficient transcription of a subgenomic RNA that encodes the viral CP and its subsequent efficient translation^7,8^. An *A. tumefaciens*-delivered TMV vector, in which most of the viral CP open reading frame (ORF) is replaced by the cDNA encoding the protein of interest, was recently developed and allowed for a high-yield production of an antifungal peptide in infiltrated *Nicotiana benthamiana* tissue^9^. Interestingly, the fusion of a signal peptide from a rice protein, which targeted the recombinant protein for export, ameliorated the toxic effect of the recombinant protein in plant tissues and facilitated purification by simple extraction of apoplastic fluid^9^.

Xylan, together with cellulose and lignin, is one of the major structural polymers of plants, representing around 20–40% of their biomass. Xylan is composed of a backbone of xylose units linked by β-1,4 bonds and branched with side chains of arabinofuranose and glucuronic acid. The precise composition and abundance of these side chains are highly variable, depending on the plant species from which the xylan derives. Due to its nature and abundance, xylan is a useful compound for many different industries, including those involving pulp and paper, biofuels, food and beverages, and animal feed^10–13^. Xylanases have relevant industrial applications; for instance, biobleaching in pulp treatment in paper industry^14,15^, or the hydrolysis of polysaccharides into simple sugars (2 to 6 unit xylooligosaccharides; XOS) with important functional properties, such as prebiotics or low-calorie sweeteners^16–23^. The use of xylanases in industrial processes is often carried out under extreme pH and temperature conditions that conventional enzymes cannot withstand. Therefore, enzymes active under such extreme conditions have been identified and isolated^24,25^. Extremophilic xylanases are particularly useful for XOS production as their operational conditions greatly favor substrate and product solubility and prevent contamination in a sugar-rich environment, otherwise prone to microbial contamination^26^.

Enzymes tailored for specific applications are often produced in large amounts in heterologous systems. The bacterium *Escherichia coli*, the yeast *Pichia pastoris*, and the filamentous fungus *Aspergillus niger* are the most frequently used organisms in industrial enzyme production. They are particularly suited due to their inexpensive culture conditions and good productivity^27^. However, the use of these microorganisms has some shortcomings. For instance, *E. coli* is unsuitable for producing secreted eukaryotic enzymes^28^. *P. pastoris* is useful for producing a number of enzymes, but the productivity is highly variable^29^. *A. niger* and other filamentous fungi are frequently restricted to homologous products, as their more complex genetics compared to *E. coli* or *P. pastoris* hampers heterologous production. In this context, plants can be a valuable alternative for heterologous production of proteins aimed to different biotechnological applications.

Since xylanases are in-demand enzymes for several industrial applications, particularly the digestion of xylan for producing XOS, in this work we analyzed whether the production of one of these enzymes in *N. benthamiana* using a TMV-derived vector and an export-to-apoplast strategy to facilitate purification is a good alternative to the classic *E. coli* host. We focused on the extremophilic xylanase Xyn11 from *Pseudothermotoga thermarum* DSM 5069^25^, an enzyme active under high temperature (90 °C) and alkaline pH (10.5), which are the most convenient conditions in an industrial setting.

## Results

### TMV-based production of xylanase Xyn11 in *N. benthamiana*

**Figure 1:**
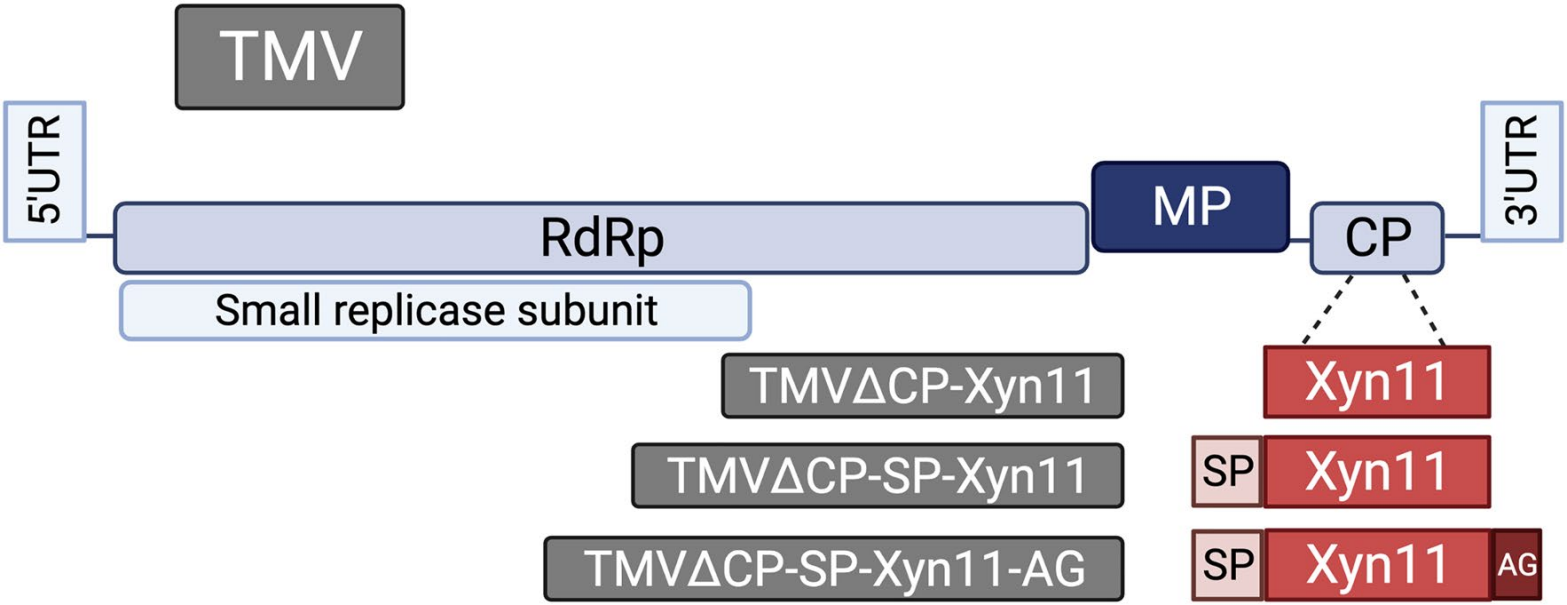
Schematic representation of TMV-derived viral vectors to express different forms of xylanase Xyn11. Boxes represent viral 5′ and 3′ untranslated regions (5′ and 3′ UTR), RNA-dependent RNA polymerase (RdRp), movement protein (MP), coat protein (CP), xylanase Xyn11 (Xyn11), (1–3)-β-endoglucanase signal peptide (SP) and arabinogalactan (AG) glycosylation module.

To evaluate the feasibility of producing recombinant xylanase Xyn11 using plants as biofactory, we first built a series of TMV-derived vectors to express this enzyme in *N. benthamiana*. The vectors were designed to express the xylanase *Xyn11* coding ORF with codons optimized for *N. benthamiana* (Fig. S1), under the control of TMV CP promoter. In these constructs, heterologous cDNAs replaced most of the viral CP gene (TMVΔCP), which generates viral vectors that can move from cell-to-cell, but not long distance through the inoculated plants^9^. Three different vectors were built, to produce xylanase Xyn11 alone for cytosolic accumulation (TMVΔCP-Xyn11), xylanase Xyn11 with an amino-terminal signal peptide (TMVΔCP-SP-Xyn11) to target the recombinant protein to the export pathway and accumulation in apoplastic space^9^, and the former with a carboxy-terminal peptide for arabinogalactan glycosylation (TMVΔCP-SP-Xyn11-AG) (Fig. 1; Fig. S2). We chose the signal peptide of the *N. tabacum* (1–3)-β-endoglucanase for its reported efficient apoplast targeting in *N. benthamiana*^30^. We also observed the effect of fusing an arabinogalactan-protein module to the carboxy-terminal end, due to reported beneficial effects on protein accumulation in tobacco cells^31^. The resulting plasmids (pTMVΔCP-Xyn11, pTMVΔCP-SP-Xyn11 and pTMVΔCP-SP-Xyn11-AG) were used to electroporate *A. tumefaciens*. The cultures of transformed *A. tumefaciens* clones were used to infiltrate leaves of *N. benthamiana* plants. The infiltrated tissues were harvested 7 days post-inoculation (dpi). Protein extracts from whole *N. benthamiana* tissues or from apoplastic liquid were analyzed by denaturing polyacrylamide gel electrophoresis (PAGE), and the gels were stained with Coomassie brilliant blue.

**Figure 2:**
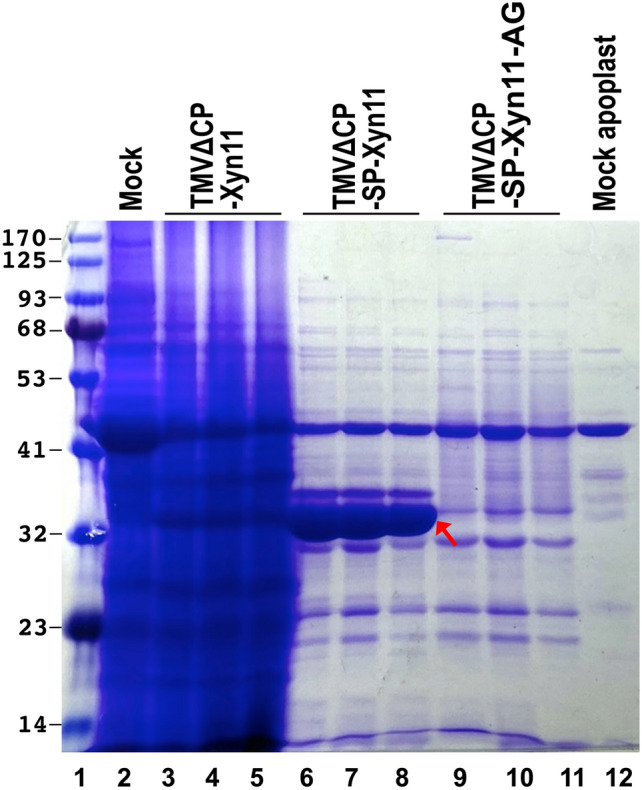
Electrophoretic analysis of protein extracts from *N. benthamiana* leaves infiltrated with different viral vectors. Leaves were harvested at 7 dpi and protein extracts were prepared from whole tissues or after recovery of apoplastic liquid. Proteins were separated by SDS-PAGE, and the gel was stained with Coomassie brilliant blue. Lane 1, marker proteins (BlueStar prestained protein marker, Nippon Genetics) with size in kDa on the left; lanes 2 to 5, total proteins from a mock-inoculated plant (lane 2) or three independent plants inoculated with TMV-Xyn11 (lane 3 to 5); lanes 6 to 12, proteins from apoplastic liquid recovered from three plants inoculated with TMV-SP-Xyn11 (lanes 6 to 8), with TMV-SP-Xyn11-AG (lanes 9 to 11), or one plant mock-inoculated (lane 12). Position of recombinant xylanase Xyn11 is indicated by a red arrow.

The results demonstrated an excellent accumulation of Xyn11 in the apoplastic fluid of the infiltrated leaves at 7 dpi. Samples from the tissues inoculated with TMVΔCP-Xyn11 showed an intracellular accumulation of a protein whose migration, slightly above the 32 kDa marker, matched that expected for the recombinant protein (Fig. 2, lanes 3 to 5). The corresponding band was absent in a mock-inoculated control (Fig. 2, lane 2). The same band, exhibiting an extraordinary intensity, was also observed in the samples corresponding to the apoplastic liquid of leaves infiltrated with TMVΔCP-SP-Xyn11 (Fig. 2, lanes 6 to 8, red arrow). No intense band was observed in the apoplast samples from the leaves infiltrated with TMVΔCP-SP-Xyn11-AG (Fig. 2, lanes 9 to 11). A band with such an intensity was also absent in the apoplastic control from a mock-inoculated plant (Fig. 2, lane 12). Taken together, these results support that the expression of Xyn11 with an export signal peptide in *N. benthamiana* using the TMVΔCP-SP-Xyn11 vector may be an excellent alternative to *E. coli* for the recombinant production of this enzyme of industrial interest.

Harvest time and the effect of an RNA silencing suppressor, namely tomato bushy stunt virus (TBSV) p19, were analyzed in the case of the TMVΔCP-SP-Xyn11 vector. Analyses confirmed 7 dpi as an optimum harvest time of plant tissues and also indicated a positive effect of p19 co-expression on Xyn11 accumulation (Fig. S3). Based on denaturing PAGE analysis with bovine serum albumin (BSA) standards followed by Coomassie brilliant blue staining, Xyn11 yield was estimated 0.68 ± 0.01 and 0.83 ± 0.04 µg/µl apoplastic fluid without and with p19 co-expression, respectively (Fig. S4). Considering 1 ml of apoplstic fluid is approximately recovered per 1 g of leaf tissue (fresh weight), these correspond to 0.68 ± 0.01 and 0.83 ± 0.04 µg Xyn11 per 1 mg N*. benthamiana* leaf tissue.

### Comparative analysis of Xyn11 produced in *E. coli* and *N. benthamiana*

**Figure 3:**
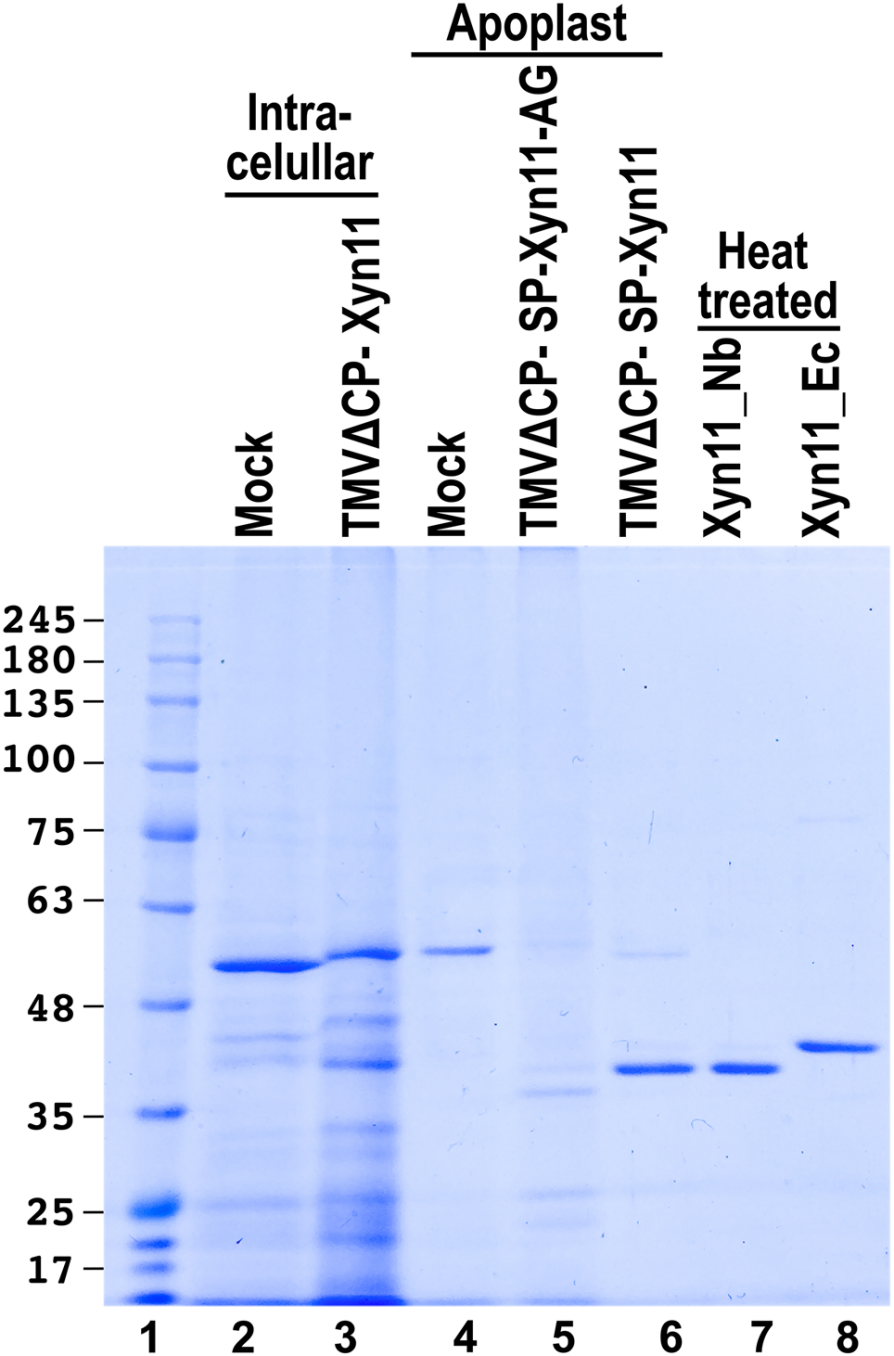
Comparative analysis of recombinant xylanases produced in *N. benthamiana* and *E. coli*. Lane 1, protein markers with size in kDa on the left; lanes 2 and 3, intracellular proteins from *N. benthamiana* mock-inoculated (lane 2) and inoculated with TMVΔCP-Xyn11 (lane 3); lanes 4 to 6, apoplastic proteins from *N. benthamiana* mock inoculated (lane 4) and inoculated with TMVΔCP-SP-Xyn11-AG (lane 5) and TMVΔCP-SP-Xyn11 (lane 6). Lanes 7 and 8, heat-treated preparations of xylanase Xyn11 purified from *N. benthamiana* apoplast (Xyn11_Nb) and *E. coli* (Xyn11_Ec).

Xylanase Xyn11 is produced in *E. coli* with a high yield: up to 20 mg of protein per liter of culture can be recovered^25^. The thermoresistant nature of the protein makes purification easy to a high degree by subjecting the bacterial cell extract to heating for several minutes. The electrophoretic analysis of Xyn11 protein produced in *N. benthamiana* and *E. coli* (hereafter Xyn11_Nb and Xyn11_Ec, respectively; Fig. 3) reveals differences in the molecular mass due to differences in the genetic constructs used for expression in these two organisms. The protein produced in the bacteria contains an N-terminal extension of 20 residues, which includes a poly-His tail added to the sequence to facilitate the purification of the protein by Ni affinity chromatography, and a C-terminal extension of 9 residues from the cloning vector^25^ (Fig. S1). The molecular masses corresponding to the amino acid composition of Xyn11_Nb and Xyn11_Ec are 40.02 and 43.17 kDa respectively, which fits well with the migration of the proteins by SDS-PAGE (Fig. 3, lanes 7 and 8). This result suggests that, in *N. benthamiana*, Xyn11 is not subjected to post-translational modifications*,* or at least these are not significant enough to change the electrophoretic migration.

**Figure 4:**
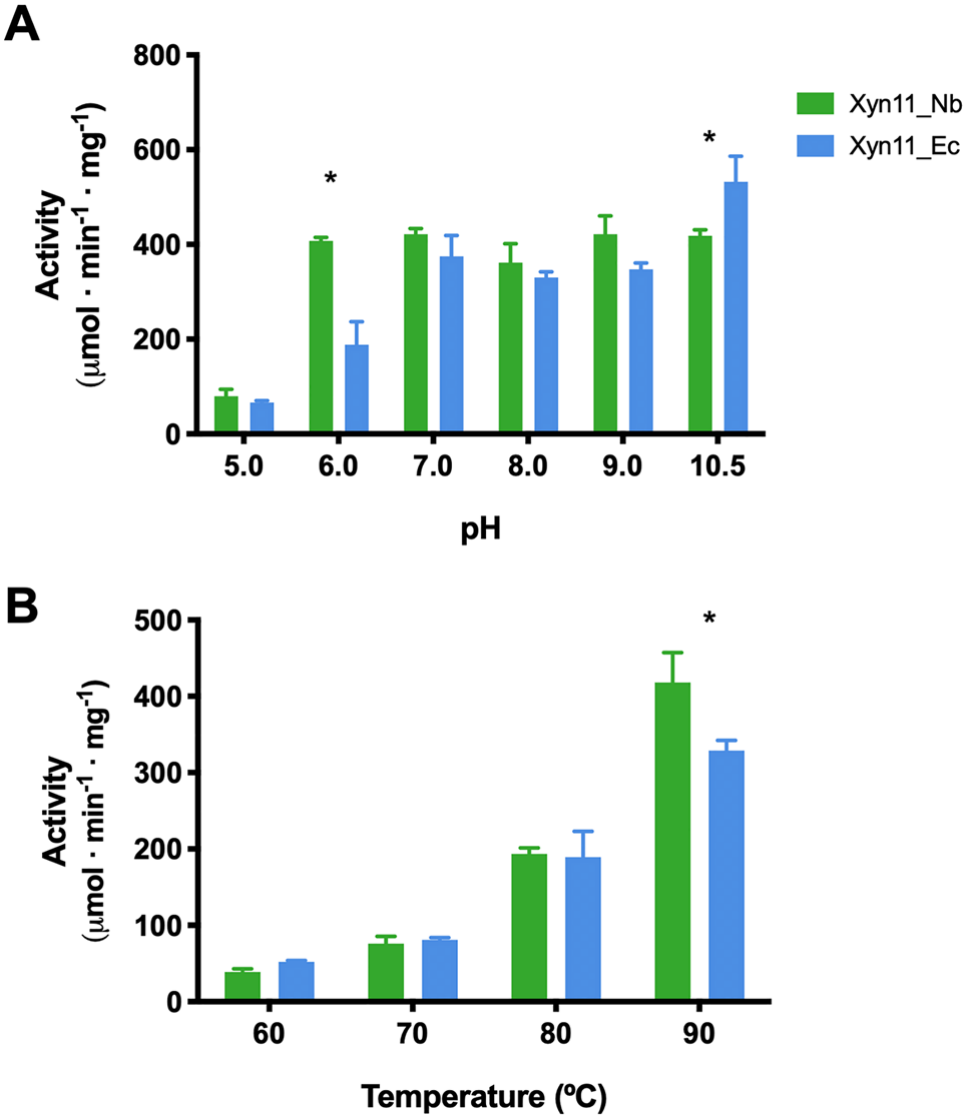
Comparative xylanolytic activity of the xylanase Xyn11 produced in *N. benthamiana* (Xyn11_Nb) and *E. coli* (Xyn11_Ec). Activity was determined at different values of (**A**) pH or (**B**) temperature, as indicated. Histograms represent the average of triplicate measurements. Error bars represent standard deviation. Conditions of pH or temperature in which both recombinant enzymes exhibit a catalytic significant difference (p < 0.01) are indicated by asterisks.

**Figure 5:**
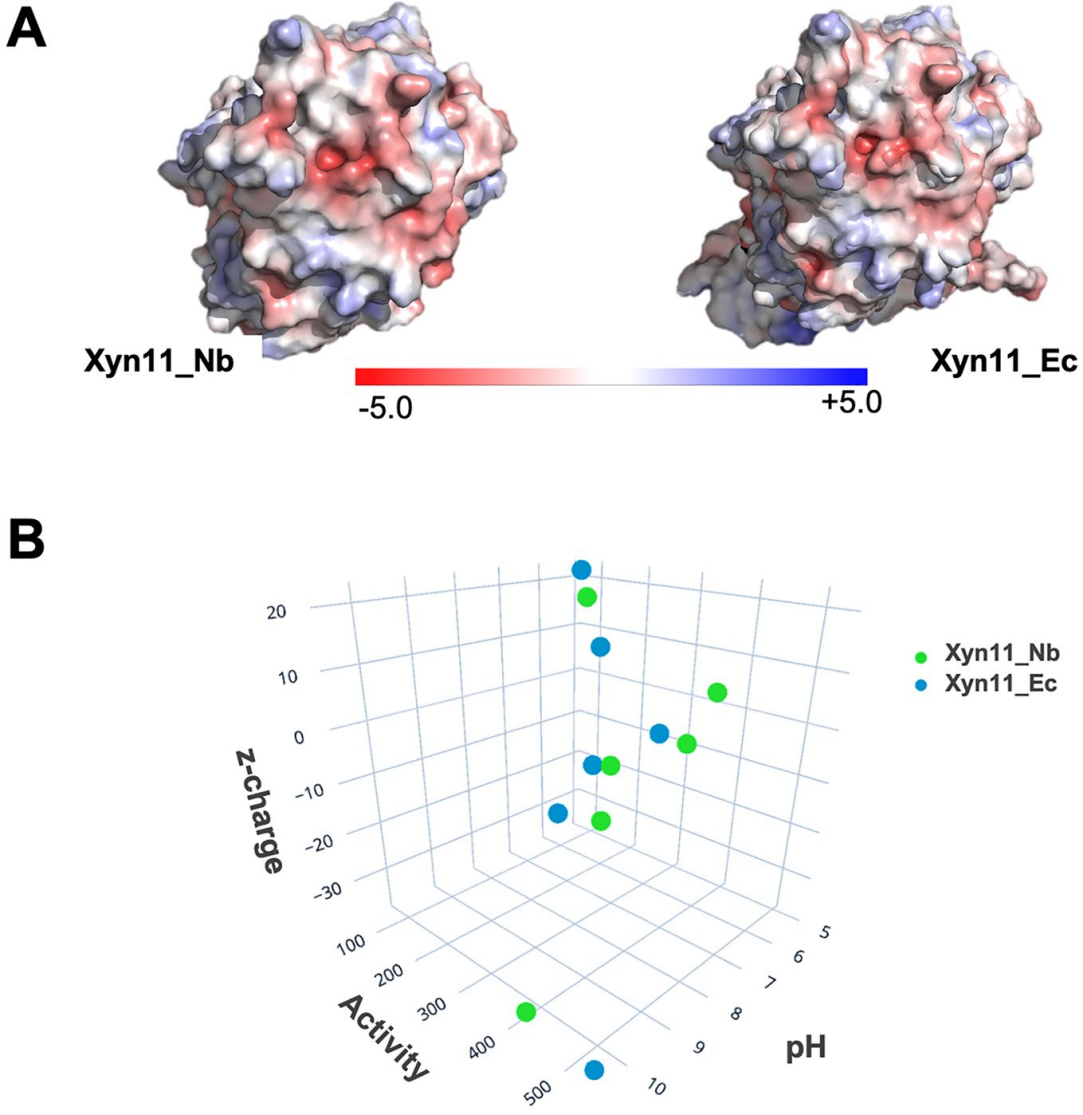
Comparative structure and pH dependent activity of the xylanase Xyn11 produced in *N. benthamiana* (Xyn11_Nb) and *E. coli* (Xyn11_Ec). (**A**) Charged surface potential representation of models of the two proteins based on the crystallographic structure of Xyn11 (PDB id: 7NL2), calculated using APBS electrostatic software (https://www.poissonboltzmann.org) and rendered with Pymol 2.3.4 (https://pymol.org/). (**B**) 3D scatter plot representation of xylanase activity and protein net charge (Z) as a function of pH variation.

The enzymatic (xylanolytic) activity of the proteins produced in the two hosts was compared under different conditions of pH and temperature. Assays at different pH, carried out at 90 °C (Fig. 4A) showed that Xyn11_Nb and Xyn11_Ec have rather similar behavior at different pH values. Both enzymes exhibited high activity in alkaline conditions (pH 7.0 to 10.5) and lower activity in acidic conditions (pH 5.0). Remarkably, there are significant differences at pH levels of 6.0 and 10.5. At pH 6.0, Xyn11_Nb doubles the activity of Xyn11_Ec, whereas at pH 10.5, Xyn11_Ec activity is significantly higher (ca. 15%) than Xyn11_Nb. These differences may be due to the presence of supernumerary ionizable residues at the N-terminal and C-terminal regions of Xyn11_Ec, which are not present in Xyn11_Nb. These might influence the protein structure, changing the stability and activity of the enzyme. Assays at different temperatures, carried out a pH 9.0 (Fig. 4B) were equivalent for both enzymes, except at the highest temperature (90 °C) where Xyn11_Nb showed higher activity than Xyn11_Ec, possibly also because of the aforementioned differences related to the presence of ionizable residues at the termini of the Xyn11_Ec sequence. Further analysis was carried out to explain the pH-dependent variation of activity observed for the two enzymes (Fig. 5). Differences in the protein structures of Xyn11_Nb and Xyn11_Ec, and particularly differences in acidic (blue) and basic (red) ionizable residues (Fig. 5A), must account for significant differences in activity at some pH values. We analyzed the interdependence of the three variables, activity, pH and protein net charge (Table S1). Results showed that activity increases at negative values of net charge, being low at relatively high values of positive net charge (Fig. 5B). This may explain the observed difference at pH 6.0 (Fig. 4A), likely due to the presence of the poly-His tail at the N-terminus of Xyn11_Ec.

Overall, the two enzymes display excellent activity profiles at high temperatures and an alkaline pH. The structural stability of the enzyme is an important asset from an industrial point of view, as it implies an extended lifetime. In addition, enzyme operation under extreme conditions drastically reduces the risk of microbial contamination. The enzyme version produced in plants shows two important additional advantages. Being secreted to the apoplast, it can be easily recovered without cell disruption, which is required to extract the enzyme produced intracellularly in *E. coli*. Furthermore, the use of a plant host is preferable to *E. coli*, particularly for food-related biotechnological applications, when considering regulatory aspects and consumer perceptions.

### Use of Xyn11 for the production of XOS

**Figure 6:**
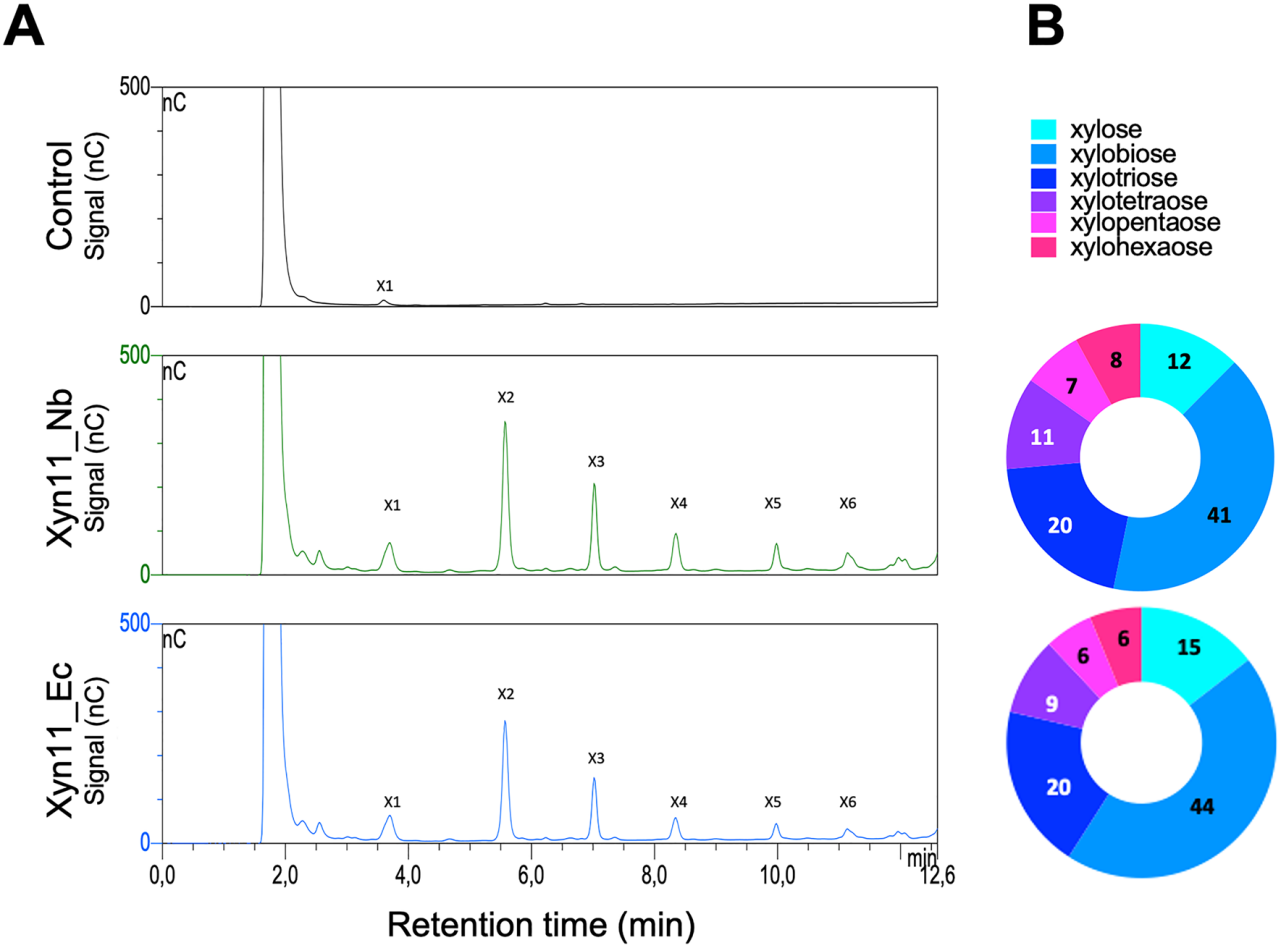
Enzymatic production of XOS. (**A**) Chromatographic profile of XOS obtained by hydrolysis of xylan using Xyn11 produced in *N. benthamiana* (Xyn11_Nb) or *E. coli* (Xyn11_Ec)*.* (**B**) Percentage of xylose and XOS obtained after enzymatic reaction with Xyn11_Nb and Xyn11_Ec.

Production of XOS from beechwood xylan was studied with Xyn11_Ec and Xyn11_Nb at pH 9.0 and at 90 °C (reaction kinetics reported in Fig. S5). The chromatographic profile of the products recovered after 2 h of reaction showed equivalent result for both enzymes (Fig. 6). Xylose and XOS (2 to 6 units) were recovered in accordance with the endo-acting mechanism of the enzyme^25^. The chromatographic profiles of the reaction products obtained with both enzymes showed relatively higher abundances of xylobiose and xylotriose (370 mg and 150 mg XOS per gram beechwood xylan, respectively), which is important since these compounds have higher prebiotic activity whereas the presence of xylose is undesirable^32^. Xylan was largely converted into 2 to 6 unit XOS (830 mg XOS/g of xylan).

## Discussion

In this work, a combination of a TMV-derived vector^9^ and *N. benthamiana* as a host plant, as well as the addition of the efficient signal peptide derived from *N. tabacum* (1–3)-β-endoglucanase^30^ that targets the recombinant protein to the apoplast, results in a remarkable accumulation of the enzyme Xyn11 in the extracellular space of plant tissues (Fig. 2). Interestingly, this production yield, in terms of the mass of *N. benthamiana* leaves or liters of *E. coli* culture to be processed can be considered comparable (Fig. 3). More specifically, in our experimental conditions for recombinant Xyn11, we estimated 20 mg per liter of *E. coli* culture and 830 mg per kg (fresh weight) of *N. benthamiana* leaves.

To date, the production of bacterial xylanases in plants for further purification and industrial use has not been largely explored. Most efforts have been directed to the expression of glycosyl hydrolases for in situ plant cell wall degradation and biofuel production^33–35^. Plant virus-derived vectors have been used for this purpose. Endoglucanase E1 and xylanase Xyn10A from *Acidothermus cellulolyticus* were transiently expressed in sunflower leaves using vacuum infiltration of *A. tumefaciens* cultures^36^. Three types of constructs were assayed, two of them consisting of viral vectors derived from cucumber mosaic virus (genus *Cucumomirus*, family *Bromoviridae*) and TMV. In contrast to our results, this work reported that virus-driven production of the cell wall-degrading enzymes was unsuccessful. Efficient expression was achieved using a cauliflower mosaic virus (CaMV) 35S promoter, and an improvement was accomplished by using methyl jasmonic acid in the agroinfiltration buffer and by optimizing the leaf incubation temperature^36^. A pepper mottle virus (genus *Potyvirus*) vector was also used to produce the endoglucanase D from *Clostridium cellulovorans* in *N. benthamiana*^37^. This work shows the feasibility of using plant virus vectors to systemically express bacterial enzymes to digest the cellulose of plant cells for biomass production. The production yield was much lower than reported here, which is likely related to the different capacities to produce heterologous proteins from tobamovirus and potyvirus vectors. Notably, a thermostable xylanase was efficiently produced in the chloroplasts of *N. tabacum* with no impact on photosynthetic performance of transplastomic plants^38^. Multi-organelle targeting was also used to improve xylanase accumulation in plant cells^39^.

As we show in this report, the enzymatic properties of Xyn11_Nb and Xyn11_Ec are similar, particularly those related to the activity under the high temperature and pH conditions required in industrial applications (Fig. 4) and the specific products of reaction (Fig. 6). However, the production of Xyn11 in plants presents a series of advantages. First, Xyn11_Nb can be tagged as a “green” product that may be a preferred option for many consumers. Second, scaling production in plants to reach the demand peaks is easier and cheaper, compared to processes based on *E. coli* fermentation. Third, the export-to-the-apoplast strategy followed by heat treatment (Figs. 2, 3) avoids cumbersome chromatographic purification of cellular extracts. This also facilitates the expression of the native amino acid sequence with no extra tags for protein purification. These tags, such as the His_6_ and linkers used in Xyn11_Ec, may modify enzymatic properties and stability (Fig. 4). Post-purification tag removal, although possible, is undesirable due to the additional processing and production costs.

Xyn11_Nb produced 2 to 6 unit XOS, yielding up to 830 mg per gram of beechwood xylan. This yield is higher than that previously reported^19^, using a crude xylanase extract from *Aureobasidium pullulans* (170.8 ± 4.0 mg XOS/g xylan). It is also higher than that reported for a thermostable xylanase from the fungus *Myceliophthora heterothallica* (234.2 mg XOS/g beechwood xylan)^40^. A yield equivalent to what we report in this communication were obtained with a commercial enzymatic cocktail^41^, and with an extremophilic xylanase from *Thermotoga thermarum*^42^. In addition to their prebiotic function, XOS have other properties that make them suitable food ingredients. For example, they can be added to cheese, giving it the characteristics of a full-fat product. They also have cryoprotective properties due to their interaction with proteins that prevent denaturation during frozen storage^43^. Xyn11 high yield production in the plant system together with the enzyme properties represent important assets for eventual uses in the food industry.

In conclusion, xylanase Xyn11 is efficiently produced in *N. benthamiana* plants using TMV as the expression vector. The purification of the recombinant protein produced in plants is highly facilitated by targeting the enzyme to the apoplast. The plant-produced Xyn11 showed excellent catalytic activity in alkaline pH and high temperature conditions, similar to the counterpart version produced in *E. coli*. However, in contrast to the bacterial version of Xyn11, the plant enzyme shows significantly higher activity at acidic pH. Remarkably, the plant enzyme exhibits higher catalytic activity than the bacterial counterpart at 90 °C and pH 9.0, which are favorable reaction conditions for industrial enzymatic xylan hydrolysis. Based on the price-effective scalability of recombinant protein production in plants and the current demand of plant-derived (green) products in food industry and other sectors, Xyn11_Nb represents an attractive alternative for enzymatic xylan hydrolysis in industrial processes.

## Materials and methods

### Plasmid construction for xylanase expression in *N. benthamiana*

The DNA sequence encoding Xyn11 (GenBank accession no. AEH51686.1) was edited to optimize codon usage in *N. benthamiana* (Fig. S1) and obtained by gene synthesis (IDT). cDNAs corresponding to Xyn11 and derivatives with N-terminal signal peptide (SP-Xyn11) and a C-terminal peptide module for arabinogalactan glycosylation (SP-Xyn11-AG) (Fig. S2) were amplified by the polymerase chain reaction (PCR) using the Phusion high-fidelity DNA polymerase in HF buffer (Thermo Scientific) and specific primers (Table S2). Reaction conditions consisted of an initial denaturation at 98 °C for 30 s, followed by 30 cycles of 10 s at 98ºC, 30 s at 55 °C and 1 min at 72 °C, and a final extension at 72 °C for 10 min. cDNAs were inserted into the plasmid pMTMVi-N^9^, opened by digestion with *Age*I and *Xho*I (Thermo Scientific). The plasmid assembly was performed using the NEBuilder HiFi DNA assembly master mix (New England Biolabs). *Bsa*I fragments from the resulting plasmids were assembled into pGTMV (containing a TMV infectious clone, GenBank accession number MK087763.1) digested with *Nco*I and *Pfl*23II (Thermo Scientific)^9^ to build pGTMVΔCP-Xyn11, pGTMVΔCP-SP-Xyn11 and pGTMVΔCP-SP-Xyn11-AG (Fig. 1 and Fig. S2).

### Agroinoculation of *N. benthamiana* leaves

The strain GV3101:pMP90 of *A. tumefaciens*, which harbors the helper plasmid pCLEAN-S48^44^, was electroporated with the plasmids pGTMVΔCP-Xyn11, pGTMVΔCP-SP-Xyn11, and pGTMVΔCP-SP-Xyn11-AG. The transformed colonies were selected in plates with a lysogenic broth (LB) medium containing 50 μg/ml rifampicin, 50 μg/ml kanamycin, and 7.5 μg/ml tetracycline. Liquid cultures were grown for 24 h at 28 °C in the same medium. Bacteria were recovered via centrifugation and brought to an optical density at 600 nm of 0.5 in agroinoculation solution (10 mM MES-NaOH, pH 5.6, 10 mM MgCl_2_ and 150 μM acetosyringone). The cultures were incubated for 2 h at 28 °C to induce the virulence genes, and used to infiltrate the leaves of 5-week old *N. benthamiana* plants. *A. tumefaciens* cultures were infiltrated at the abaxial side of the leaves using a needleless 1-mL syringe. The infiltrated plants were cultured in a growth chamber at 25ºC with a 12-h day-night photoperiod. In some experiments, leaves were co-agroinfiltrated with 1:1 mixes of *A. tumefaciens* transformed with pGTMVΔCP-SP-Xyn11 and a plasmid to express TBSV p19 RNA silencing suppressor under the control of CaMV 35S promoter and terminator or the corresponding empty plasmid. Each *A. tumefaciens* culture was brought to an optical density of 1.0 and induced as stated above, and mixed 1:1 previously to infiltration. Experimental research on *N. benthamiana* complied with relevant institutional, national, and international guidelines and legislation, and permissions were obtained.

### Production and purification of Xyn11 in* E. coli*

The Xyn11 sequence was identified in a computational analysis, where xylanases active under extreme conditions of temperature and alkaline pH were searched for. A synthetic DNA fragment coding for the protein sequence, optimized for translation in *E. coli*, was cloned in an expression vector as previously described^25^. Cell extracts from the *E. coli* cultures expressing a His-tagged version of Xyn11, prepared in 20 mM phosphate buffer, pH 7.4, 10 mM imidazole, 500 mM NaCl, were heated at 85 °C for 10 min, which caused the precipitation of most of the proteins, leaving the xylanase in a clarified supernatant after centrifugation. The enzyme preparation was subjected to Ni-affinity chromatography in a 1 mL HisTrap FF column (Cytiva) mounted in an AKTA-Purifier (Cytiva). Elution was carried out with a 20 mM phosphate buffer, pH 7.4, 500 mM imidazole, 500 mM NaCl. This version of the enzyme was termed Xyn11_Ec.

### Purification of Xyn11 from* N. benthamiana* leaves

Infiltrated tissues from *N. benthamiana* plants were harvested at different dpi, as indicated. For total protein extraction, tissues were ground with a mortar and pestle in the presence of liquid N_2_. Three volumes of the buffer TEW (60 mM Tris–HCl, pH 6.8, 2% sodium dodecyl sulphate [SDS], 100 mM dithiothreitol, 10% glycerol and 0.01% bromophenol blue) were added. The extracts were incubated for 5 min at 95 °C and clarified by centrifugation for 15 min. For apoplastic liquid recovery, the tissues were first vacuum-infiltrated with phosphate buffered saline (PBS) buffer supplemented with 0.02% (v/v) Silwet L-77, then introduced in a 50 mL syringe and squeezed. Finally, the apoplastic fractions were clarified by centrifugation for 5 min at 13,000 rpm. This version of the enzyme was termed Xyn11_Nb.

### Enzyme analysis

The activity of the purified xylanases (Xyn11_Ec and Xyn11_Nb) was assayed at a range of high temperatures and alkaline pH. Enzymatic reactions at different temperatures (60, 70, 80, or 90 °C) were carried out by mixing 180 μL beechwood xylan 1% (Megazyme) with 20 μL of the enzyme in a Tris–HCl buffer (50 mM) at a pH of 9.0. The enzyme concentration was adjusted to assure a lineal response. Analyses at different pH values were carried out at 90 °C, using 50 mM buffered solutions at the following pH: 5.0 (acetate), 6.0 and 7.0 (phosphate), 8.0, 9.0 (Tris–HCl), and 10.5 (CAPS). Reactions were terminated by placing the tubes on ice. To measure sugar reduction resulting from xylan digestion, 100 μL of dinitro salicylic acid (DNS) solution was added to the reaction tubes, which were subsequently boiled for 10 min. After boiling, 900 μL of miliQ H_2_O was added, and the tubes were centrifuged. A fraction of the supernatant (300 μL) from each reaction tube was transferred to a 96-well plate, and optical density at 540 nm (OD_540_) was measured using a PowerWave HT spectrophotometer, from BioTek Instruments (Winooski, VT, USA). Statistical analysis was performed by two-way ANOVA, using a *p* value < 0.001.

### Xylan digestion and XOS analysis

The production of XOS was carried out using as a substrate commercial beechwood xylan (Megazyme) at a 1% concentration in a 50 mM Tris–HCl buffer, pH 9.0. Xylanases, Xyn11_Ec or Xyn11_Nb, were added at 0.75 µg/mL and incubated at 90ºC for different times. The reactions were stopped by placing the tubes on ice. XOS were analyzed by exchange ion chromatography, using a DIONEX instrument equipped with a CarboPac PA100 column and a pulsed amperometric detector (Dionex, Thermo Fisher Scientific). Xylose (Sigma-Aldrich) and xylooligosaccharides, from two to six units (Megazyme), were used as the chromatographic standards.

## Supplementary Information

Supplementary Information 1.

Supplementary Information 2.

Supplementary Information 3.

Supplementary Information 4.

## Data Availability

Relevant DNA sequences handled in this work are deposited in GenBank under accession numbers AEH51686.1 and MK087763.1.
