# Supplementary material for: Expression of an extremophilic xylanase in Nicotiana benthamiana and its use for the production of prebiotic xylooligosaccharides
Source: Sci Rep. 2022 Sep 21;12:15743. doi: 10.1038/s41598-022-19774-5 (PMC9492658; doi:10.1038/s41598-022-19774-5)
Supplement: Supplementary file 1 — Supplementary Information 1. [file 41598_2022_19774_MOESM1_ESM.pdf]

SUPPLEMENTARY MATERIAL

Expression of an extremophilic xylanase in *Nicotiana benthamiana* and its use for the production of prebiotic xylooligosaccharides

David Talens-Perales[1,a], María Nicolau-Sanus[2,a], Julio Polaina[1,*], José-Antonio Daròs[2,*]

David Talens-Perales, https://orcid.org/0000-0002-8693-4239

María Nicolau-Sanus, https://orcid.org/0000-0002-9963-5131

Julio Polaina, https://orcid.org/0000-0001-9912-0640

José-Antonio Daròs, https://orcid.org/0000-0002-6535-2889

[1]Department of Food Biotechnology. Institute of Agrochemistry and Food Technology, Spanish National Research Council (IATA-CSIC), Paterna, Valencia, Spain. [2]Instituto de Biología Molecular y Ceular de Plantas (Consejo Superior de Investigaciones Científicas-Universitat Politècnica de València), 46022 Valencia, Spain. [a]David Talens-Perales and María Nicolau-Sanus contributed equally to this work. *email: jpolaina@iata.csic.es; jadaros@ibmcp.upv.es

Fig. S1. Nucleotide and amino acid sequences of the xylanase Xyn11 versions expressed in *Escherichia coli* (Xyn11_Ec) and *Nicotiana benthamiana* (Xyn11_Nb).

>Xyn11_Ec

```
ATGAGAGGATCGCATCACCATCACCATCACGGATCCGCATGCGAGCTCGGGCCTCAGCCGATGTCGTCGCAAATC
CCCTCACTGAAGGATGTCTTTCTTCAAGATTTCAAAATCGGCGTGGCACTGCCGGTACGCGTCTTCTCTAATTCC
ATGGATGTTGAGTTAATTACCAAACATTTCAATTCGATGACAGCGGAAAATGAGATGAAGCCCGAATCGATTTTA
CGTCGTGATGCCTCGGGGAAGATTTATTATGACTTCACCGTTGCTGACCGCTACATCGAATTTGCACAGAAGCAT
GGCATGGTGGTACGCGGACACACATTAGTTTGGCACTCCCAAACACCGGAATGGTTTTTCAAAGATGAAAAGGGC
AATTTATTGTCGCGTGAAGCAATGATTGAACGTATGCGCGAATACATCCACACAGTAGTAGGCCGCTACCGTGGG
AAAGTTTACGCTTGGGACGTCGTTAATGAAGCCGTGGACGAAAACCAGCCAGACGGACTGCGTCGTTCGTTATGG
TATCAGGTTATCGGCCCAGATTATATTGAGCTTGCATTTAAGTTTGCGCATGAGGCTGACCCAGATGCATTGTTA
TTTTACAACGACTATAATGAATTCTTTCCGAAAAAACGTGACATCATCTTTAAGTTGGTTAAGGAGATGCGCGAA
AAGGGCGTCCCAATTCATGGGATCGGAATGCAACAGCATCTTACACTTGCGGACAACGTGGGGTGGATCGATATC
GCAATCCAAAAATTTAAGACTATCAGTGGGATCCAAATCCATATTACGGAACTTGACGTATCTGTCTACAAGAGT
CGTTCTCCAAGCATTATTTATCAGACACCTCCACTTGAAGTGCTTAAAGAACAAGCGGAATTTTATCGTAAACTT
TTTGAGATTTATCGCAAGCACACCGACGTAATTACCAATGTTACATTTTGGGGGTTGAAGGACGACTACAGCTGG
TTGCGTTTCTTTTTTGGCCGTCGCAACGACTGGCCGCTGCTTTTTGACGAGAATTATCAACCGAAACCTGCGTTT
TGGAGTGTTATCGAATCAGTAAGCAAGGTCGACCTGCAGCCAAGCTTAATTAGCTGA
```

>Xyn11_Ec

MRGSHHHHHHGSACELGPQPMSSQIPSLKDVFLQDFKIGVALPVRVFSNSMDVELITKHFNSMTAENEMKPESIL
RRDASGKIYYDFTVADRYIEFAQKHGMVVRGHTLVWHSQTPEWFFKDEKGNLLSREAMIERMREYIHTVVGRYRG
KVYAWDVVNEAVDENQPDGLRRSLWYQVIGPDYIELAFKFAHEADPDALLFYNDYNEFFPKKRDIIFKLVKEMRE
KGVPIHGIGMQQHLTLADNVGWIDIAIQKFKTISGIQIHITELDVSVYKSRSPSIIYQTPPLEVLKEQAEFYRKL
FEIYRKHTDVITNVTFWGLKDDYSWLRFFFGRRNDWPLLFDENYQPKPAFWSVIESVSKVDLQPSLIS*

Original **Xyn11** sequence in black, **His$_6$** tag from pQE80L (*E. coli* expression vector) in red, and **other amino acids**, besides the His$_6$ tag, from pQE80L in blue.

>Xyn11_Nb

```
ATGAGTTCTCAAATACCTTCTTTAAAAGATGTGTTCCTTCAGGATTTTAAGATCGGAGTCGCCCTGCCCGTGCGA
GTCTTCTCTAACTCAATGGACGTCGAGCTGATAACGAAACATTTTAACAGCATGACGGCAGAGAACGAGATGAAA
CCTGAGAGTATTCTGAGGCGAGACGCAAGTGGAAAGATATATTACGACTTTACAGTCGCTGATAGGTATATTGAG
TTTGCTCAGAAACATGGCATGGTCGTTCGAGGCCATACCCTGGTCTGGCATTCACAGACGCCCGAATGGTTTTTT
AAAGATGAAAAGGGTAACTTGTTGTCACGAGAAGCTATGATTGAGAGGATGCGTGAATATATTCATACCGTTGTC
GGTAGATACAGGGGTAAAGTATACGCCTGGGATGTTGTCAATGAAGCAGTTGATGAAAATCAACCAGATGGGTTA
AGAAGGTCCCTTTGGTATCAGGTAATCGGGCCCGACTACATTGAGTTGGCCTTTAAATTCGCTCACGAAGCTGAC
CCTGATGCACTGCTTTTCTATAACGACTACAATGAATTCTTCCCGAAAAAGAGAGATATCATATTTAAGCTTGTT
AAAGAAATGAGGGAAAAGGGGGTGCCAATACATGGTATTGGAATGCAGCAGCACTTGACACTTGCTGATAACGTA
GGTTGGATTGACATAGCCATTCAGAAATTTAAAACGATCAGTGGCATCCAGATTCATATAACAGAACTGGATGTA
TCAGTCTACAAAAGCCGTTCTCCAAGTATTATATACCAGACCCCGCCTTTAGAAGTTTTGAAAGAACAAGCCGAA
TTTTATCGAAAGTTATTCGAGATTTACAGGAAGCATACGGACGTCATCACCAATGTGACGTTCTGGGGATTGAAG
GACGACTACAGCTGGTTGAGATTCTTCTTTGGCAGAAGAAATGATTGGCCCCTGTTGTTCGACGAGAACTATCAG
CCTAAGCCGGCTTTTTGGTCCGTCATAGAGTCTGTATCAAAATGA
```

>Xyn11_Nb

MSSQIPSLKDVFLQDFKIGVALPVRVFSNSMDVELITKHFNSMTAENEMKPESILRRDASGKIYYDFTVADRYIE
FAQKHGMVVRGHTLVWHSQTPEWFFKDEKGNLLSREAMIERMREYIHTVVGRYRGKVYAWDVVNEAVDENQPDGL
RRSLWYQVIGPDYIELAFKFAHEADPDALLFYNDYNEFFPKKRDIIFKLVKEMREKGVPIHGIGMQQHLTLADNV
GWIDIAIQKFKTISGIQIHITELDVSVYKSRSPSIIYQTPPLEVLKEQAEFYRKLFEIYRKHTDVITNVTFWGLK
DDYSWLRFFFGRRNDWPLLFDENYQPKPAFWSVIESVSK*

Fig. S2. Sequence of TMV recombinant clones to express different versions of xylanase Xyn11 in *N. benthamiana* (Xyn11_Nb). Vectors were built on the basis of a TMV infectious variant (GenBank accession no. MK087763.1) with deletion of most of CP gene and mutation of viral CP initiation ATG to **AGA** (in red). Nucleotide sequence of **Xyn11_Nb** is in blue, **(1-3)-β-endoglucanase signal peptide (SP)** in purple, and the **arabinogalactan (AG) glycosylation module** in gold colors.

>TMVΔCP-Xyn11

GTATTTTTACAACAATTACCAACAACAACAAACAACAAACAACATTACAATTACTATTTACAATTACAATGGCAT
ACACACAGACAGCTACCACATCAGCTTTGCTGGACACTGTCCGAGGAAACAACTCCTTGGTCAATGATCTAGCAA
AGCGTCGTCTTTACGACACAGCGGTTGAAGAGTTTAACGCTCGTGACCGCAGGCCCAAGGTGAACTTTTCAAAAG
TAATAAGCGAGGAGCAGACGCTTATTGCTACCCGGGCGTATCCAGAATTCCAAATTACATTTTATAACACGCAAA
ATGCCGTGCATTCGCTTGCAGGTGGATTGCGATCTTTAGAACTGGAATATCTGATGATGCAAATTCCCTACGGAT
CATTGACTTATGACATAGGCGGGAATTTTGCATCGCATCTGTTCAAGGGACGAGCATATGTACACTGCTGCATGC
CCAACCTGGACGTTCGAGACATCATGCGGCACGAAGGCCAGAAAGACAGTATTGAACTATACCTTTCTAGGCTAG
AGAGAGGGGGGAAAACAGTCCCCAACTTCCAAAAGGAAGCATTTGACAGATACGCAGAAATTCCTGAAGACGCTG
TCTGTCACAATACTTTCCAGACATGCGAACATCAGCCGATGCAGCAATCAGGCAGAGTGTATGCCATTGCGCTAC
ACAGCATATATGACATACCAGCCGATGAGTTCGGGGCGGCACTCTTGAGGAAAAATGTCCATACGTGCTATGCCG
CTTTCCACTTCTCCGAGAACCTGCTTCTTGAAGATTCATGCGTCAATTTGGACGAAATCAACGCGTGTTTTTCGC
GCGATGGAGACAAGTTGACCTTTTCTTTTGCATCAGAGAGTACTCTTAATTACTGTCATAGTTATTCTAATATTC
TTAAGTATGTGTGCAAAACTTACTTCCCGGCCTCTAATAGAGAGGTTTACATGAAGGAGTTTTTAGTCACCAGAG
TTAATACCTGGTTTTGTAAGTTTTCTAGAATAGATACTTTTCTTTTGTACAAAGGTGTGGCCCATAAAAGTGTAG
ATAGTGAGCAGTTTTATACTGCAATGGAAGACGCATGGCATTACAAAAAGACTCTTGCAATGTGCAACAGCGAGA
GAATCCTCCTTGAGGATTCATCATCAGTCAATTACTGGTTTCCCAAAATGAGGGATATGGTCATCGTACCATTAT
TCGACATTTCTTTGGAGACTAGTAAGAGGACGCGCAAGGAAGTCTTAGTGTCCAAGGATTTCGTGTTTACAGTGC
TTAACCACATTCGAACATACCAGGCGAAAGCTCTTACATACGCAAATGTTTTGTCCTTCGTCGAATCGATTCGAT
CGAGGGTAATCATTAACGGTGTGACAGCGAGGTCCGAATGGGATGTGGACAAATCTTTGTTACAATCCTTGTCCA
TGACGTTTTACCTGCATACTAAGCTTGCCGTTCTAAAGGATGACTTACTGATTAGCAAGTTTAGTCTCGGTTCGA
AAACGGTGTGCCAGCATGTGTGGGATGAGATTTCGCTGGCGTTTGGGAACGCATTTCCCTCCGTGAAAGAGAGGC
TCTTGAACAGGAAACTTATCAGAGTGGCAGGCGACGCATTAGAGATCAGGGTGCCTGATCTATATGTGACCTTCC
ACGACAGATTAGTGACTGAGTACAAGGCCTCTGTGGACATGCCTGCGCTTGACATTAGGAAGAAGATGGAAGAAA
CGGAAGTGATGTACAATGCACTTTCAGAATTATCGGTGTTAAGGGAGTCTGACAAATTCGATGTTGATGTTTTTT
CCCAGATGTGCCAATCTTTGGAAGTTGACCCAATGACGGCAGCGAAGGTTATAGTCGCGGTCATGAGCAATGAGA
GCGGTCTGACTCTCACATTTGAACGACCTACTGAGGCGAATGTTGCGCTAGCTTTACAGGATCAAGAGAAGGCTT
CAGAAGGTGCATTGGTAGTTACCTCAAGAGAAGTTGAAGAACCGTCCATGAAGGGTTCGATGGCCAGAGGAGAGT
TACAATTAGCTGGTCTTGCTGGAGATCATCCGGAGTCGTCCTATTCTAAGAACGAGGAGATAGAGTCTTTAGAGC
AGTTTCATATGGCGACGGCAGATTCGTTAATTCGTAAGCAGATGAGCTCGATTGTGTACACGGGTCCGATTAAAG
TTCAGCAAATGAAAAACTTTATCGATAGCCTGGTAGCATCACTATCTGCTGCGGTGTCGAATCTCGTCAAGATCC
TCAAAGATACAGCTGCTATTGACCTTGAAACCCGTCAAAAGTTTGGAGTCTTGGATGTTGCATCTAGGAAGTGGT
TAATCAAACCAACGGCCAAGAGTCATGCATGGGGTGTTGTTGAAACCCACGCGAGGAAGTATCATGTGGCGCTTT
TGGAATATGATGAGCAGGGTGTGGTGACATGCGATGATTGGAGAAGAGTAGCTGTTAGCTCTGAGTCTGTTGTTT
ATTCCGACATGGCGAAACTCAGAACTCTGCGCAGACTGCTTCGAAACGGAGAACCGCATGTCAGTAGCGCAAAGG
TTGTTCTTGTGGACGGAGTTCCGGGCTGTGGAAAAACCAAAGAAATTCTTTCCAGGGTTAATTTTGATGAAGATC
TAATTTTAGTACCTGGGAAGCAAGCCGCGGAAATGATCAGAAGACGTGCGAATTCCTCAGGGATTATTGTGGCCA
CGAAGGACAACGTTAAAACCGTTGATTCTTTCATGATGAATTTTGGGAAAAGCACACGCTGTCAGTTCAAGAGGT
TATTCATTGATGAAGGGTTGATGTTGCATACTGGTTGTGTTAATTTTCTTGTGGCGATGTCATTGTGCGAAATTG
CATATGTTTACGGAGACACACAGCAGATTCCATACATCAATAGAGTTTCAGGATTCCCGTACCCCGCCCATTTTG
CCAAATTGGAAGTTGACGAGGTGGAGACACGCAGAACTACTCTCCGTTGTCCAGCCGATGTCACACATTATCTGA
ACAGGAGATATGAGGGCTTTGTCATGAGCACTTCTTCGGTTAAAAAGTCTGTTTCGCAGGAGATGGTCGGCGGAG
CCGCCGTGATCAATCCGATCTCAAAACCCTTGCATGGCAAGATCCTGACTTTTACCCAATCGGATAAAGAAGCTC
TGCTTTCAAGAGGGTATTCAGATGTTCACACTGTGCATGAAGTGCAAGGCGAGACATACTCTGATGTTTCACTAG
TTAGGTTAACCCCTACACCGGTCTCCATCATTGCAGGAGACAGCCCACATGTTTTGGTCGCATTGTCAAGGCACA
CCTGTTCGCTCAAGTACTACACTGTTGTTATGGATCCTTTAGTTAGTATCATTAGAGATCTAGAGAAACTTAGCT
CGTACTTGTTAGATATGTATAAGGTCGATGCAGGAACACAATAGCAATTACAGATTGACTCGGTGTTCAAAGGTT
CCAATCTTTTTGTTGCAGCGCCAAAGACTGGTGATATTTCTGATATGCAGTTTTACTATGATAAGTGTCTCCCAG
GCAACAGCACCATGATGAATAATTTTGATGCTGTTACCATGAGGTTGACTGACATTTCATTGAATGTCAAAGATT
GCATATTGGATATGTCTAAGTCTGTTGCTGCGCCTAAGGATCAAATCAAACCACTAATACCTATGGTACGAACGG
CGGCAGAAATGCCACGCCAGACTGGACTATTGGAAAATTTAGTGGCGATGATTAAAAGAAACTTTAACGCACCCG
AGTTGTCTGGCATCATTGATATTGAAAATACTGCATCTTTGGTTGTAGATAAGTTTTTTGATAGTTATTTGCTTA
AGAAAAAAAGAAAACCAAATAAAAATGTTTCTTTGTTCAGTAGAGAGTCTCTCAATAGATGGTTAGAAAAGCAGG

AACAGGTAACAATAGGCCAGCTCGCAGATTTTGATTTTGTGGATTTGCCAGCAGTTGATCAGTACAGACACATGA
TTAAAGCACAACCCAAACAAAAGTTGGACACTTCAATCCAAACGGAGTACCCGGCTTTGCAGACGATTGTGTACC
ATTCAAAAAAGATCAATGCAATATTCGGCCCGTTGTTTAGTGAGCTTACTAGGCAATTACTGGACAGTGTTGATT
CGAGCAGATTTTTGTTTTTCACAAGAAAGACACCAGCGCAGATTGAGGATTTCTTCGGAGATCTCGACAGTCATG
TGCCGATGGATGTCTTGGAGCTGGATATATCAAAATACGACAAATCTCAGAATGAATTCCACTGTGCAGTAGAAT
ACGAGATCTGGCGAAGATTGGGTTTTGAAGACTTCTTGGGAGAAGTTTGGAAACAAGGGCATAGAAAGACCACCC
TCAAGGATTATACCGCAGGTATAAAAACTTGCATCTGGTATCAAAGAAAGAGCGGGGACGTCACGACGTTCATTG
GAAACACTGTGATCATTGCTGCATGTTTGGCCTCGATGCTTCCGATGGAGAAAATAATCAAAGGAGCCTTTTGCG
GTGACGATAGTCTGCTGTACTTTCCAAAGGGTTGTGAGTTTCCGGATGTGCAACACTCCGCAAATCTTATGTGGA
ATTTTGAAGCAAAACTGTTTAAAAAACAGTATGGATACTTTTGCGGAAGATATGTAATACATCACGACAGAGGAT
GCATTGTGTATTACGATCCCCTAAAGTTGATCTCGAAACTTGGTGCTAAACACATCAAGGATTGGGAACACTTGG
AGGAGTTCAGAAGGTCTCTTTGTGATGTTGCTGTTTCGTTGAACAATTGTGCGTATTACACACAGTTGGACGACG
CTGTATGGGAGGTTCATAAGACCGCCCCTCCAGGTTCGTTTGTTTATAAAAGTCTGGTGAAGTATTTGTCTGATA
AAGTTCTTTTTAGAAGTTTGTTTATAGATGGCTCTAGTTGTTAAAGGAAAAGTGAATATCAATGAGTTTATCGAC
CTGTCAAAAATGGAGAAGATCTTACCGTCGATGTTTACCCCTGTAAAGAGTGTTATGTGTTCCAAAGTTGATAAA
ATAATGGTTCATGAGAATGAGTCATTGTCAGAGGTGAACCTTCTTAAAGGAGTTAAGCTTATTGATAGTGGATAC
GTCTGTTTAGCCGGTTTGGTCGTCACGGGCGAGTGGAACTTGCCTGACAATTGCAGAGGAGGTGTGAGCGTGTGT
CTGGTGGACAAAAGGATGGAAAGAGCCGACGAGGCCACTCTCGGATCTTACTACACAGCAGCTGCAAAGAAAAGA
TTTCAGTTCAAGGTCGTTCCCAATTATGCTATAACCACCCAGGACGCGATGAAAAACGTCTGGCAAGTTTTAGTT
AATATTAGAAATGTGAAGATGTCAGCGGGTTTCTGTCCGCTTTCTCTGGAGTTTGTGTCGGTGTGTATTGTTTAT
AGAAATAATATAAAATTAGGTTTGAGAGAGAAGATTACAAACGTGAGAGACGGAGGGCCCATGGAACTTACAGAA
GAAGTCGTTGATGAGTTCATGGAAGATGTCCCTATGTCGATCAGGCTTGCAAAGTTTCGATCTCGAACCGGAAAA
AAGAGTGATGTCCGCAAAGGGAAAAATAGTAGTAGTGATCGGTCAGTGCCGAACAAGAACTATAGAAATGTTAAG
GATTTTGGAGGAATGAGTTTTAAAAAGAATAATTTAATCGATGATGATTCGGAGGCTACTGTCGCCGAATCGGAT
TCGTTTTAAAT**AGA**TCTTACAGTATCACTACTCCATCTCAGTTCGTGTTCTTGTC**ATGAGTTCTCAAATACCTTC**
TTTAAAAGATGTGTTCCTTCAGGATTTTAAGATCGGAGTCGCCCTGCCCGTGCGAGTCTTCTCTAACTCAATGGA
CGTCGAGCTGATAACGAAACATTTTAACAGCATGACGGCAGAGAACGAGATGAAACCTGAGAGTATTCTGAGGCG
AGACGCAAGTGGAAAGATATATTACGACTTTACAGTCGCTGATAGGTATATTGAGTTTGCTCAGAAACATGGCAT
GGTCGTTCGAGGCCATACCCTGGTCTGGCATTCACAGACGCCCGAATGGTTTTTAAAGATGAAAAGGGTAACTT
GTTGTCACGAGAAGCTATGATTGAGAGGATGCGTGAATATATTCATACCGTTGTCGGTAGATACAGGGGTAAAGT
ATACGCCTGGGATGTTGTCAATGAAGCAGTTGATGAAAATCAACCAGATGGGTTAAGAAGGTCCCTTTGGTATCA
GGTAATCGGGCCCGACTACATTGAGTTGGCCTTTAAATTCGCTCACGAAGCTGACCCTGATGCACTGCTTTTCTA
TAACGACTACAATGAATTCTTCCCGAAAAAGAGAGATATCATATTTAAGCTTGTTAAAGAAATGAGGGAAAAGGG
GGTGCCAATACATGGTATTGGAATGCAGCAGCACTTGACACTTGCTGATAACGTAGGTTGGATTGACATAGCCAT
TCAGAAATTTAAAACGATCAGTGGCATCCAGATTCATATAACAGAACTGGATGTATCAGTCTACAAAAGCCGTTC
TCCAAGTATTATATACCAGACCCCGCCTTTAGAAGTTTTGAAAGAACAAGCCGAATTTTATCGAAAGTTATTCGA
GATTTACAGGAAGCATACGGACGTCATCACCAATGTGACGTTCTGGGGATTGAAGGACGACTACAGCTGGTTGAG
ATTCTTCTTTGGCAGAAGAAATGATTGGCCCCTGTTGTTCGACGAGAACTATCAGCCTAAGCCGGCTTTTTGGTC
CGTCATAGAGTCTGTATCAAAATGAGGTCCTGCAACTTGAGGTAGTCAAGATGCATAATAAATAACGGATTGTGT
CCGTAATCACACGTGGTGCGTACGATAACGCATAGTGTTTTTCCCTCCACTTAAATCGAAGGGTTGTGTCTTGGA
TCGCGCGGGTCAAATGTATATGGTTCATATACATCCGCAGGCACGTAATAAAGCGAGGGGTTCGAATCCCCCCGT
TACCCCCGGTAGGGGCCCA

>TMVΔCP-SP-Xyn11
GTATTTTTACAACAATTACCAACAACAACAAACAACAAACAACATTACAATTACTATTTACAATTACAATGGCAT
ACACACAGACAGCTACCACATCAGCTTTGCTGGACACTGTCCGAGGAAACAACTCCTTGGTCAATGATCTAGCAA
AGCGTCGTCTTTACGACACAGCGGTTGAAGAGTTTAACGCTCGTGACCGCAGGCCCAAGGTGAACTTTTCAAAAG
TAATAAGCGAGGAGCAGACGCTTATTGCTACCCGGGCGTATCCAGAATTCCAAATTACATTTTATAACACGCAAA
ATGCCGTGCATTCGCTTGCAGGTGGATTGCGATCTTTAGAACTGGAATATCTGATGATGCAAATTCCCTACGGAT
CATTGACTTATGACATAGGCGGGAATTTTGCATCGCATCTGTTCAAGGGACGAGCATATGTACACTGCTGCATGC
CCAACCTGGACGTTCGAGACATCATGCGGCACGAAGGCCAGAAAGACAGTATTGAACTATACCTTTCTAGGCTAG
AGAGAGGGGGGAAAACAGTCCCCAACTTCCAAAAGGAAGCATTTGACAGATACGCAGAAATTCCTGAAGACGCTG
TCTGTCACAATACTTTCCAGACATGCGAACATCAGCCGATGCAGCAATCAGGCAGAGTGTATGCCATTGCGCTAC
ACAGCATATATGACATACCAGCCGATGAGTTCGGGGCGGCACTCTTGAGGAAAAATGTCCATACGTGCTATGCCG
CTTTCCACTTCTCCGAGAACCTGCTTCTTGAAGATTCATGCGTCAATTTGGACGAAATCAACGCGTGTTTTTCGC
GCGATGGAGACAAGTTGACCTTTTCTTTTGCATCAGAGAGTACTCTTAATTACTGTCATAGTTATTCTAATATTC
TTAAGTATGTGTGCAAAACTTACTTCCCGGCCTCTAATAGAGAGGTTTACATGAAGGAGTTTTTAGTCACCAGAG
TTAATACCTGGTTTTGTAAGTTTTCTAGAATAGATACTTTTCTTTTGTACAAAGGTGTGGCCCATAAAAGTGTAG
ATAGTGAGCAGTTTTATACTGCAATGGAAGACGCATGGCATTACAAAAAGACTCTTGCAATGTGCAACAGCGAGA
GAATCCTCCTTGAGGATTCATCATCAGTCAATTACTGGTTTCCCAAAATGAGGGATATGGTCATCGTACCATTAT
TCGACATTTCTTTGGAGACTAGTAAGAGGACGCGCAAGGAAGTCTTAGTGTCCAAGGATTTCGTGTTTACAGTGC

TTAACCACATTCGAACATACCAGGCGAAAGCTCTTACATACGCAAATGTTTTGTCCTTCGTCGAATCGATTCGAT
CGAGGGTAATCATTAACGGTGTGACAGCGAGGTCCGAATGGGATGTGGACAAATCTTTGTTACAATCCTTGTCCA
TGACGTTTTACCTGCATACTAAGCTTGCCGTTCTAAAGGATGACTTACTGATTAGCAAGTTTAGTCTCGGTTCGA
AAACGGTGTGCCAGCATGTGTGGGATGAGATTTCGCTGGCGTTTGGGAACGCATTTCCCTCCGTGAAAGAGAGGC
TCTTGAACAGGAAACTTATCAGAGTGGCAGGCGACGCATTAGAGATCAGGGTGCCTGATCTATATGTGACCTTCC
ACGACAGATTAGTGACTGAGTACAAGGCCTCTGTGGACATGCCTGCGCTTGACATTAGGAAGAAGATGGAAGAAA
CGGAAGTGATGTACAATGCACTTTCAGAATTATCGGTGTTAAGGGAGTCTGACAAATTCGATGTTGATGTTTTTT
CCCAGATGTGCCAATCTTTGGAAGTTGACCCAATGACGGCAGCGAAGGTTATAGTCGCGGTCATGAGCAATGAGA
GCGGTCTGACTCTCACATTTGAACGACCTACTGAGGCGAATGTTGCGCTAGCTTTACAGGATCAAGAGAAGGCTT
CAGAAGGTGCATTGGTAGTTACCTCAAGAGAAGTTGAAGAACCGTCCATGAAGGGTTCGATGGCCAGAGGAGAGT
TACAATTAGCTGGTCTTGCTGGAGATCATCCGGAGTCGTCCTATTCTAAGAACGAGGAGATAGAGTCTTTAGAGC
AGTTTCATATGGCGACGGCAGATTCGTTAATTCGTAAGCAGATGAGCTCGATTGTGTACACGGGTCCGATTAAAG
TTCAGCAAATGAAAAACTTTATCGATAGCCTGGTAGCATCACTATCTGCTGCGGTGTCGAATCTCGTCAAGATCC
TCAAAGATACAGCTGCTATTGACCTTGAAACCCGTCAAAAGTTTGGAGTCTTGGATGTTGCATCTAGGAAGTGGT
TAATCAAACCAACGGCCAAGAGTCATGCATGGGGTGTTGTTGAAACCCACGCGAGGAAGTATCATGTGGCGCTTT
TGGAATATGATGAGCAGGGTGTGGTGACATGCGATGATTGGAGAAGAGTAGCTGTTAGCTCTGAGTCTGTTGTTT
ATTCCGACATGGCGAAACTCAGAACTCTGCGCAGACTGCTTCGAAACGGAGAACCGCATGTCAGTAGCGCAAAGG
TTGTTCTTGTGGACGGAGTTCCGGGCTGTGGAAAAACCAAAGAAATTCTTTCCAGGGTTAATTTTGATGAAGATC
TAATTTTAGTACCTGGGAAGCAAGCCGCGGAAATGATCAGAAGACGTGCGAATTCCTCAGGGATTATTGTGGCCA
CGAAGGACAACGTTAAAACCGTTGATTCTTTCATGATGAATTTTGGGAAAAGCACACGCTGTCAGTTCAAGAGGT
TATTCATTGATGAAGGGTTGATGTTGCATACTGGTTGTGTTAATTTTCTTGTGGCGATGTCATTGTGCGAAATTG
CATATGTTTACGGAGACACACAGCAGATTCCATACATCAATAGAGTTTCAGGATTCCCGTACCCCGCCCATTTTG
CCAAATTGGAAGTTGACGAGGTGGAGACACGCAGAACTACTCTCCGTTGTCCAGCCGATGTCACACATTATCTGA
ACAGGAGATATGAGGGCTTTGTCATGAGCACTTCTTCGGTTAAAAAGTCTGTTTCGCAGGAGATGGTCGGCGGAG
CCGCCGTGATCAATCCGATCTCAAAACCCTTGCATGGCAAGATCCTGACTTTTACCCAATCGGATAAAGAAGCTC
TGCTTTCAAGAGGGTATTCAGATGTTCACACTGTGCATGAAGTGCAAGGCGAGACATACTCTGATGTTTCACTAG
TTAGGTTAACCCCTACACCGGTCTCCATCATTGCAGGAGACAGCCCACATGTTTTGGTCGCATTGTCAAGGCACA
CCTGTTCGCTCAAGTACTACACTGTTGTTATGGATCCTTTAGTTAGTATCATTAGAGATCTAGAGAAACTTAGCT
CGTACTTGTTAGATATGTATAAGGTCGATGCAGGAACACAATAGCAATTACAGATTGACTCGGTGTTCAAAGGTT
CCAATCTTTTTGTTGCAGCGCCAAAGACTGGTGATATTTCTGATATGCAGTTTTACTATGATAAGTGTCTCCCAG
GCAACAGCACCATGATGAATAATTTTGATGCTGTTACCATGAGGTTGACTGACATTTCATTGAATGTCAAAGATT
GCATATTGGATATGTCTAAGTCTGTTGCTGCGCCTAAGGATCAAATCAAACCACTAATACCTATGGTACGAACGG
CGGCAGAAATGCCACGCCAGACTGGACTATTGGAAAATTTAGTGGCGATGATTAAAAGAAACTTTAACGCACCCG
AGTTGTCTGGCATCATTGATATTGAAAATACTGCATCTTTGGTTGTAGATAAGTTTTTTGATAGTTATTTGCTTA
AAGAAAAAAGAAAACCAAATAAAAATGTTTCTTTGTTCAGTAGAGAGTCTCTCAATAGATGGTTAGAAAAGCAGG
AACAGGTAACAATAGGCCAGCTCGCAGATTTTGATTTTGTGGATTTGCCAGCAGTTGATCAGTACAGACACATGA
TTAAAGCACAACCCAAACAAAAGTTGGACACTTCAATCCAAACGGAGTACCCGGCTTTGCAGACGATTGTGTACC
ATTCAAAAAAGATCAATGCAATATTCGGCCCGTTGTTTAGTGAGCTTACTAGGCAATTACTGGACAGTGTTGATT
CGAGCAGATTTTTGTTTTTCACAAGAAAGACACCAGCGCAGATTGAGGATTTCTTCGGAGATCTCGACAGTCATG
TGCCGATGGATGTCTTGGAGCTGGATATATCAAAATACGACAAATCTCAGAATGAATTCCACTGTGCAGTAGAAT
ACGAGATCTGGCGAAGATTGGGTTTTGAAGACTTCTTGGGAGAAGTTTGGAAACAAGGGCATAGAAAGACCACCC
TCAAGGATTATACCGCAGGTATAAAAACTTGCATCTGGTATCAAAGAAAGAGCGGGGACGTCACGACGTTCATTG
GAAACACTGTGATCATTGCTGCATGTTTGGCCTCGATGCTTCCGATGGAGAAAATAATCAAAGGAGCCTTTTGCG
GTGACGATAGTCTGCTGTACTTTCCAAAGGGTTGTGAGTTTCCGGATGTGCAACACTCCGCAAATCTTATGTGGA
ATTTTGAAGCAAAACTGTTTAAAAAACAGTATGGATACTTTTGCGGAAGATATGTAATACATCACGACAGAGGAT
GCATTGTGTATTACGATCCCCTAAAGTTGATCTCGAAACTTGGTGCTAAACACATCAAGGATTGGGAACACTTGG
AGGAGTTCAGAAGGTCTCTTTGTGATGTTGCTGTTTCGTTGAACAATTGTGCGTATTACACACAGTTGGACGACG
CTGTATGGGAGGTTCATAAGACCGCCCCTCCAGGTTCGTTTGTTTATAAAAGTCTGGTGAAGTATTTGTCTGATA
AAGTTCTTTTTAGAAGTTTGTTTATAGATGGCTCTAGTTGTTAAAGGAAAAGTGAATATCAATGAGTTTATCGAC
CTGTCAAAAATGGAGAAGATCTTACCGTCGATGTTTACCCCTGTAAAGAGTGTTATGTGTTCCAAAGTTGATAAA
ATAATGGTTCATGAGAATGAGTCATTGTCAGAGGTGAACCTTCTTAAAGGAGTTAAGCTTATTGATAGTGGATAC
GTCTGTTTAGCCGGTTTGGTCGTCACGGGCGAGTGGAACTTGCCTGACAATTGCAGAGGAGGTGTGAGCGTGTGT
CTGGTGGACAAAAGGATGGAAAGAGCCGACGAGGCCACTCTCGGATCTTACTACACAGCAGCTGCAAAGAAAAGA
TTTCAGTTCAAGGTCGTTCCCAATTATGCTATAACCACCCAGGACGCGATGAAAAACGTCTGGCAAGTTTTAGTT
AATATTAGAAATGTGAAGATGTCAGCGGGTTTCTGTCCGCTTTCTCTGGAGTTTGTGTCGGTGTGTATTGTTTAT
AGAAATAATATAAAATTAGGTTTGAGAGAGAAGATTACAAACGTGAGAGACGGAGGGCCCATGGAACTTACAGAA
GAAGTCGTTGATGAGTTCATGGAAGATGTCCCTATGTCGATCAGGCTTGCAAAGTTTCGATCTCGAACCGGAAAA
AAGAGTGATGTCCGCAAAGGGAAAAATAGTAGTAGTGATCGGTCAGTGCCGAACAAGAACTATAGAAATGTTAAG
GATTTTGGAGGAATGAGTTTTAAAAAGAATAATTTAATCGATGATGATTCGGAGGCTACTGTCGCCGAATCGGAT
TCGTTTTAAAT**AGA**TCTTACAGTATCACTACTCCATCTCAGTTCGTGTTCTTGTC**ATGGCTTTGTGGTACTTGTT**
CAATAAAAGGAGTCTTGGTGCAGCTGTCCTTATACTTGTTGGGCTGTTAATGTGCAATATTCAAATGACAGGAGC

TATGAGTTCTCAAATACCTTCTTTAAAAGATGTGTTCCTTCAGGATTTTAAGATCGGAGTCGCCCTGCCCGTGCG
AGTCTTCTCTAACTCAATGGACGTCGAGCTGATAACGAAACATTTTAACAGCATGACGGCAGAGAACGAGATGAA
ACCTGAGAGTATTCTGAGGCGAGACGCAAGTGGAAAGATATATTACGACTTTACAGTCGCTGATAGGTATATTGA
GTTTGCTCAGAAACATGGCATGGTCGTTCGAGGCCATACCCTGGTCTGGCATTCACAGACGCCCGAATGGTTTTT
TAAAGATGAAAAGGGTAACTTGTTGTCACGAGAAGCTATGATTGAGAGGATGCGTGAATATATTCATACCGTTGT
CGGTAGATACAGGGGTAAAGTATACGCCTGGGATGTTGTCAATGAAGCAGTTGATGAAAATCAACCAGATGGGTT
AAGAAGGTCCCTTTGGTATCAGGTAATCGGGCCCGACTACATTGAGTTGGCCTTTAAATTCGCTCACGAAGCTGA
CCCTGATGCACTGCTTTTCTATAACGACTACAATGAATTCTTCCCGAAAAAGAGAGATATCATATTTAAGCTTGT
TAAAGAAATGAGGGAAAAGGGGGTGCCAATACATGGTATTGGAATGCAGCAGCACTTGACACTTGCTGATAACGT
AGGTTGGATTGACATAGCCATTCAGAAATTTAAAACGATCAGTGGCATCCAGATTCATATAACAGAACTGGATGT
ATCAGTCTACAAAAGCCGTTCTCCAAGTATTATATACCAGACCCCGCCTTTAGAAGTTTTGAAAGAACAAGCCGA
ATTTTATCGAAAGTTATTCGAGATTTACAGGAAGCATACGGACGTCATCACCAATGTGACGTTCTGGGGATTGAA
GGACGACTACAGCTGGTTGAGATTCTTCTTTGGCAGAAGAAATGATTGGCCCCTGTTGTTCGACGAGAACTATCA
GCCTAAGCCGGCTTTTTGGTCCGTCATAGAGTCTGTATCAAAATGAGGTCCTGCAACTTGAGGTAGTCAAGATGC
ATAATAAATAACGGATTGTGTCCGTAATCACACGTGGTGCGTACGATAACGCATAGTGTTTTTCCCTCCACTTAA
ATCGAAGGGTTGTGTCTTGGATCGCGCGGGTCAAATGTATATGGTTCATATACATCCGCAGGCACGTAATAAAGC
GAGGGGTTCGAATCCCCCCGTTACCCCCGGTAGGGGCCCA

>TMVΔCP-SP-Xyn11-AG
GTATTTTTACAACAATTACCAACAACAACAAACAACAAACAACATTACAATTACTATTTACAATTACAATGGCAT
ACACACAGACAGCTACCACATCAGCTTTGCTGGACACTGTCCGAGGAAACAACTCCTTGGTCAATGATCTAGCAA
AGCGTCGTCTTTACGACACAGCGGTTGAAGAGTTTAACGCTCGTGACCGCAGGCCCAAGGTGAACTTTTCAAAAG
TAATAAGCGAGGAGCAGACGCTTATTGCTACCCGGGCGTATCCAGAATTCCAAATTACATTTTATAACACGCAAA
ATGCCGTGCATTCGCTTGCAGGTGGATTGCGATCTTTAGAACTGGAATATCTGATGATGCAAATTCCCTACGGAT
CATTGACTTATGACATAGGCGGGAATTTTGCATCGCATCTGTTCAAGGGACGAGCATATGTACACTGCTGCATGC
CCAACCTGGACGTTCGAGACATCATGCGGCACGAAGGCCAGAAAGACAGTATTGAACTATACCTTTCTAGGCTAG
AGAGAGGGGGAAAACAGTCCCCAACTTCCAAAAGGAAGCATTTGACAGATACGCAGAAATTCCTGAAGACGCTG
TCTGTCACAATACTTTCCAGACATGCGAACATCAGCCGATGCAGCAATCAGGCAGAGTGTATGCCATTGCGCTAC
ACAGCATATATGACATACCAGCCGATGAGTTCGGGGCGGCACTCTTGAGGAAAAATGTCCATACGTGCTATGCCG
CTTTCCACTTCTCCGAGAACCTGCTTCTTGAAGATTCATGCGTCAATTTGGACGAAATCAACGCGTGTTTTTCGC
GCGATGGAGACAAGTTGACCTTTTCTTTTGCATCAGAGAGTACTCTTAATTACTGTCATAGTTATTCTAATATTC
TTAAGTATGTGTGCAAAACTTACTTCCCGGCCTCTAATAGAGAGGTTTACATGAAGGAGTTTTTAGTCACCAGAG
TTAATACCTGGTTTTGTAAGTTTTCTAGAATAGATACTTTTCTTTTGTACAAAGGTGTGGCCCATAAAAGTGTAG
ATAGTGAGCAGTTTTATACTGCAATGGAAGACGCATGGCATTACAAAAAGACTCTTGCAATGTGCAACAGCGAGA
GAATCCTCCTTGAGGATTCATCATCAGTCAATTACTGGTTTCCCAAAATGAGGGATATGGTCATCGTACCATTAT
TCGACATTTCTTTGGAGACTAGTAAGAGGACGCGCAAGGAAGTCTTAGTGTCCAAGGATTTCGTGTTTACAGTGC
TTAACCACATTCGAACATACCAGGCGAAAGCTCTTACATACGCAAATGTTTTGTCCTTCGTCGAATCGATTCGAT
CGAGGGTAATCATTAACGGTGTGACAGCGAGGTCCGAATGGGATGTGGACAAATCTTTGTTACAATCCTTGTCCA
TGACGTTTTACCTGCATACTAAGCTTGCCGTTCTAAAGGATGACTTACTGATTAGCAAGTTTAGTCTCGGTTCGA
AAACGGTGTGCCAGCATGTGTGGGATGAGATTTCGCTGGCGTTTGGGAACGCATTTCCCTCCGTGAAAGAGAGGC
TCTTGAACAGGAAACTTATCAGAGTGGCAGGCGACGCATTAGAGATCAGGGTGCCTGATCTATATGTGACCTTCC
ACGACAGATTAGTGACTGAGTACAAGGCCTCTGTGGACATGCCTGCGCTTGACATTAGGAAGAAGATGGAAGAAA
CGGAAGTGATGTACAATGCACTTTCAGAATTATCGGTGTTAAGGGAGTCTGACAAATTCGATGTTGATGTTTTTT
CCCAGATGTGCCAATCTTTGGAAGTTGACCCAATGACGGCAGCGAAGGTTATAGTCGCGGTCATGAGCAATGAGA
GCGGTCTGACTCTCACATTTGAACGACCTACTGAGGCGAATGTTGCGCTAGCTTTACAGGATCAAGAGAAGGCTT
CAGAAGGTGCATTGGTAGTTACCTCAAGAGAAGTTGAAGAACCGTCCATGAAGGGTTCGATGGCCAGAGGAGAGT
TACAATTAGCTGGTCTTGCTGGAGATCATCCGGAGTCGTCCTATTCTAAGAACGAGGAGATAGAGTCTTTAGAGC
AGTTTCATATGGCGACGGCAGATTCGTTAATTCGTAAGCAGATGAGCTCGATTGTGTACACGGGTCCGATTAAAG
TTCAGCAAATGAAAAACTTTATCGATAGCCTGGTAGCATCACTATCTGCTGCGGTGTCGAATCTCGTCAAGATCC
TCAAAGATACAGCTGCTATTGACCTTGAAACCCGTCAAAAGTTTGGAGTCTTGGATGTTGCATCTAGGAAGTGGT
TAATCAAACCAACGGCCAAGAGTCATGCATGGGGTGTTGTTGAAACCCACGCGAGGAAGTATCATGTGGCGCTTT
TGGAATATGATGAGCAGGGTGTGGTGACATGCGATGATTGGAGAAGAGTAGCTGTTAGCTCTGAGTCTGTTGTTT
ATTCCGACATGGCGAAACTCAGAACTCTGCGCAGACTGCTTCGAAACGGAGAACCGCATGTCAGTAGCGCAAAGG
TTGTTCTTGTGGACGGAGTTCCGGGCTGTGGAAAAACCAAAGAAATTCTTTCCAGGGTTAATTTTGATGAAGATC
TAATTTTAGTACCTGGGAAGCAAGCCGCGGAAATGATCAGAAGACGTGCGAATTCCTCAGGGATTATTGTGGCCA
CGAAGGACAACGTTAAAACCGTTGATTCTTTCATGATGAATTTTGGGAAAAGCACACGCTGTCAGTTCAAGAGGT
TATTCATTGATGAAGGGTTGATGTTGCATACTGGTTGTGTTAATTTTCTTGTGGCGATGTCATTGTGCGAAATTG
CATATGTTTACGGAGACACACAGCAGATTCCATACATCAATAGAGTTTCAGGATTCCCGTACCCCGCCCATTTTG
CCAAATTGGAAGTTGACGAGGTGGAGACACGCAGAACTACTCTCCGTTGTCCAGCCGATGTCACACATTATCTGA
ACAGGAGATATGAGGGCTTTGTCATGAGCACTTCTTCGGTTAAAAAGTCTGTTTCGCAGGAGATGGTCGGCGGAG
CCGCCGTGATCAATCCGATCTCAAAACCCTTGCATGGCAAGATCCTGACTTTTACCCAATCGGATAAAGAAGCTC

TGCTTTCAAGAGGGTATTCAGATGTTCACACTGTGCATGAAGTGCAAGGCGAGACATACTCTGATGTTTCACTAG
TTAGGTTAACCCCTACACCGGTCTCCATCATTGCAGGAGACAGCCCACATGTTTTGGTCGCATTGTCAAGGCACA
CCTGTTCGCTCAAGTACTACACTGTTGTTATGGATCCTTTAGTTAGTATCATTAGAGATCTAGAGAAACTTAGCT
CGTACTTGTTAGATATGTATAAGGTCGATGCAGGAACACAATAGCAATTACAGATTGACTCGGTGTTCAAAGGTT
CCAATCTTTTTGTTGCAGCGCCAAAGACTGGTGATATTTCTGATATGCAGTTTTACTATGATAAGTGTCTCCCAG
GCAACAGCACCATGATGAATAATTTTGATGCTGTTACCATGAGGTTGACTGACATTTCATTGAATGTCAAAGATT
GCATATTGGATATGTCTAAGTCTGTTGCTGCGCCTAAGGATCAAATCAAACCACTAATACCTATGGTACGAACGG
CGGCAGAAATGCCACGCCAGACTGGACTATTGGAAAATTTAGTGGCGATGATTAAAAGAAACTTTAACGCACCCG
AGTTGTCTGGCATCATTGATATTGAAAATACTGCATCTTTGGTTGTAGATAAGTTTTTTGATAGTTATTTGCTTA
AAGAAAAAAGAAAACCAAATAAAAATGTTTCTTTGTTCAGTAGAGAGTCTCTCAATAGATGGTTAGAAAAGCAGG
AACAGGTAACAATAGGCCAGCTCGCAGATTTTGATTTTGTGGATTTGCCAGCAGTTGATCAGTACAGACACATGA
TTAAAGCACAACCCAAACAAAAGTTGGACACTTCAATCCAAACGGAGTACCCGGCTTTGCAGACGATTGTGTACC
ATTCAAAAAAGATCAATGCAATATTCGGCCCGTTGTTTAGTGAGCTTACTAGGCAATTACTGGACAGTGTTGATT
CGAGCAGATTTTTGTTTTTCACAAGAAAGACACCAGCGCAGATTGAGGATTTCTTCGGAGATCTCGACAGTCATG
TGCCGATGGATGTCTTGGAGCTGGATATATCAAAATACGACAAATCTCAGAATGAATTCCACTGTGCAGTAGAAT
ACGAGATCTGGCGAAGATTGGGTTTTGAAGACTTCTTGGGAGAAGTTTGGAAACAAGGGCATAGAAAGACCACCC
TCAAGGATTATACCGCAGGTATAAAAACTTGCATCTGGTATCAAAGAAAGAGCGGGGACGTCACGACGTTCATTG
GAAACACTGTGATCATTGCTGCATGTTTGGCCTCGATGCTTCCGATGGAGAAAATAATCAAAGGAGCCTTTTGCG
GTGACGATAGTCTGCTGTACTTTCCAAAGGGTTGTGAGTTTCCGGATGTGCAACACTCCGCAAATCTTATGTGGA
ATTTTGAAGCAAAACTGTTTAAAAAACAGTATGGATACTTTTGCGGAAGATATGTAATACATCACGACAGAGGAT
GCATTGTGTATTACGATCCCCTAAAGTTGATCTCGAAACTTGGTGCTAAACACATCAAGGATTGGGAACACTTGG
AGGAGTTCAGAAGGTCTCTTTGTGATGTTGCTGTTTCGTTGAACAATTGTGCGTATTACACACAGTTGGACGACG
CTGTATGGGAGGTTCATAAGACCGCCCCTCCAGGTTCGTTTGTTTATAAAAGTCTGGTGAAGTATTTGTCTGATA
AAGTTCTTTTTAGAAGTTTGTTTATAGATGGCTCTAGTTGTTAAAGGAAAAGTGAATATCAATGAGTTTATCGAC
CTGTCAAAAATGGAGAAGATCTTACCGTCGATGTTTACCCCTGTAAAGAGTGTTATGTGTTCCAAAGTTGATAAA
ATAATGGTTCATGAGAATGAGTCATTGTCAGAGGTGAACCTTCTTAAAGGAGTTAAGCTTATTGATAGTGGATAC
GTCTGTTTAGCCGGTTTGGTCGTCACGGGCGAGTGGAACTTGCCTGACAATTGCAGAGGAGGTGTGAGCGTGTGT
CTGGTGGACAAAAGGATGGAAAGAGCCGACGAGGCCACTCTCGGATCTTACTACACAGCAGCTGCAAAGAAAAGA
TTTCAGTTCAAGGTCGTTCCCAATTATGCTATAACCACCCAGGACGCGATGAAAAACGTCTGGCAAGTTTTAGTT
AATATTAGAAATGTGAAGATGTCAGCGGGTTTCTGTCCGCTTTCTCTGGAGTTTGTGTCGGTGTGTATTGTTTAT
AGAAATAATATAAAATTAGGTTTGAGAGAGAAGATTACAAACGTGAGAGACGGAGGGCCCATGGAACTTACAGAA
GAAGTCGTTGATGAGTTCATGGAAGATGTCCCTATGTCGATCAGGCTTGCAAAGTTTCGATCTCGAACCGGAAAA
AAGAGTGATGTCCGCAAAGGGAAAAATAGTAGTAGTGATCGGTCAGTGCCGAACAAGAACTATAGAAATGTTAAG
GATTTTGGAGGAATGAGTTTTAAAAAGAATAATTTAATCGATGATGATTCGGAGGCTACTGTCGCCGAATCGGAT
TCGTTTTAAAT**AGA**TCTTACAGTATCACTACTCCATCTCAGTTCGTGTTCTTGTC**ATGGCTTTGTGGTACTTGTT**
CAATAAAAGGAGTCTTGGTGCAGCTGTCCTTATACTTGTTGGGCTGTTAATGTGCAATATTCAAATGACAGGAGC
TATGAGTTCTCAAATACCTTCTTTAAAAGATGTGTTCCTTCAGGATTTTAAGATCGGAGTCGCCCTGCCCGTGCG
AGTCTTCTCTAACTCAATGGACGTCGAGCTGATAACGAAACATTTTAACAGCATGACGGCAGAGAACGAGATGAA
ACCTGAGAGTATTCTGAGGCGAGACGCAAGTGGAAAGATATATTACGACTTTACAGTCGCTGATAGGTATATTGA
GTTTGCTCAGAAACATGGCATGGTCGTTCGAGGCCATACCCTGGTCTGGCATTCACAGACGCCCGAATGGTTTTT
TAAAGATGAAAAGGGTAACTTGTTGTCACGAGAAGCTATGATTGAGAGGATGCGTGAATATATTCATACCGTTGT
CGGTAGATACAGGGGTAAAGTATACGCCTGGGATGTTGTCAATGAAGCAGTTGATGAAAATCAACCAGATGGGTT
AAGAAGGTCCCTTTGGTATCAGGTAATCGGGCCCGACTACATTGAGTTGGCCTTTAAATTCGCTCACGAAGCTGA
CCCTGATGCACTGCTTTTCTATAACGACTACAATGAATTCTTCCCGAAAAAGAGAGATATCATATTTAAGCTTGT
TAAAGAAATGAGGGAAAAGGGGGTGCCAATACATGGTATTGGAATGCAGCAGCACTTGACACTTGCTGATAACGT
AGGTTGGATTGACATAGCCATTCAGAAATTTAAAACGATCAGTGGCATCCAGATTCATATAACAGAACTGGATGT
ATCAGTCTACAAAAGCCGTTCTCCAAGTATTATATACCAGACCCCGCCTTTAGAAGTTTTGAAAGAACAAGCCGA
ATTTTATCGAAAGTTATTCGAGATTTACAGGAAGCATACGGACGTCATCACCAATGTGACGTTCTGGGGATTGAA
GGACGACTACAGCTGGTTGAGATTCTTCTTTGGCAGAAGAAATGATTGGCCCCTGTTGTTCGACGAGAACTATCA
GCCTAAGCCGGCTTTTTGGTCCGTCATAGAGTCTGTATCAAAATCACCCTCTCCAAGCCCTTCCCCATCGCCTAG
TCCCTCACCATCCCCTTCTCCCAGCCCATGAGGTCCTGCAACTTGAGGTAGTCAAGATGCATAATAAATAACGGA
TTGTGTCCGTAATCACACGTGGTGCGTACGATAACGCATAGTGTTTTTCCCTCCACTTAAATCGAAGGGTTGTGT
CTTGGATCGCGCGGGTCAAATGTATATGGTTCATATACATCCGCAGGCACGTAATAAAGCGAGGGGTTCGAATCC
CCCCGTTACCCCCGGTAGGGGCCCA

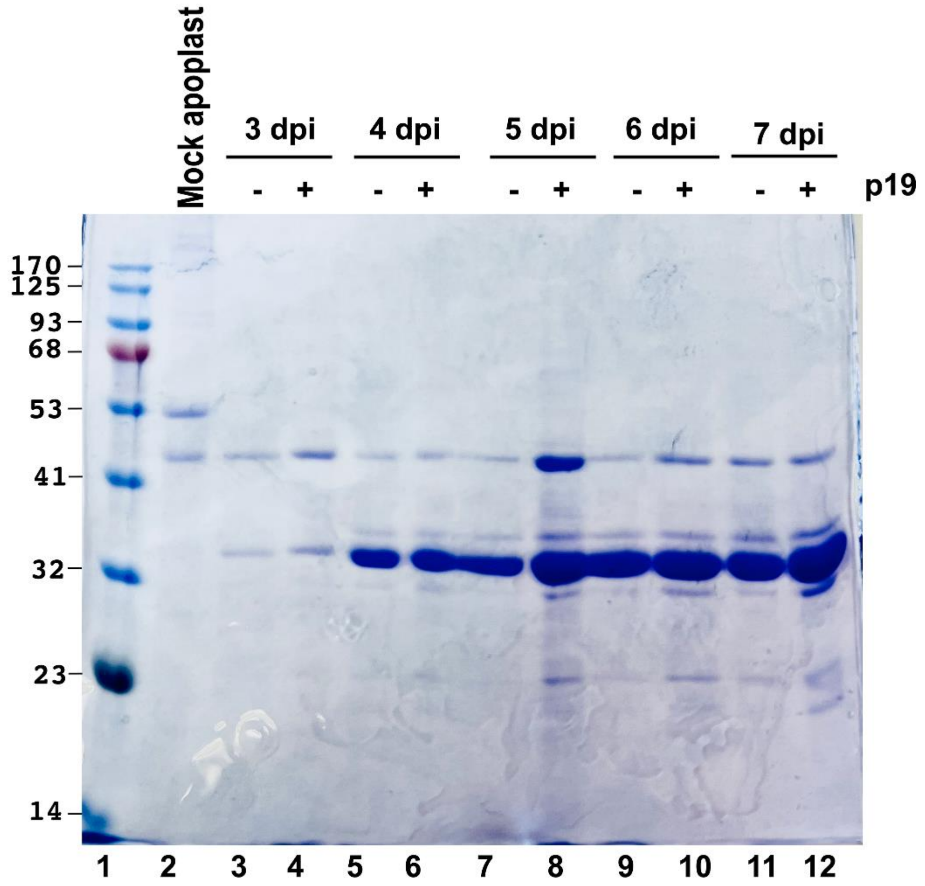

Fig. S3. Time course analysis of recombinant xylanase production in *N. benthamiana* with the viral vector TMVΔCP-SP-Xyn11 without and with co-expression of TBSV p19 RNA silencing suppressor, as indicated. Leaves were harvested from 3 to 7 dpi, as indicated, and apoplastic liquid recovered. Proteins in the apoplast were separated by SDS-PAGE, and the gel was stained with Coomassie brilliant blue. Lane 1, marker proteins with size in kDa on the left; lane 2, apoplast from a mock inoculated plant; lanes 3, 5, 7, 9 and 11, tissues harvested at 3, 4, 5, 6 and 7 dpi, respectively, from plants co-agroinoculated with TMVΔCP-SP-Xyn11 and an empty plasmid; lanes 4, 6, 8, 10 and 12, tissues harvested at 3, 4, 5, 6 and 7 dpi, respectively, from plants co-agroinoculated with TMVΔCP-SP-Xyn11 and a plasmid to express the RNA silencing suppressor p19.

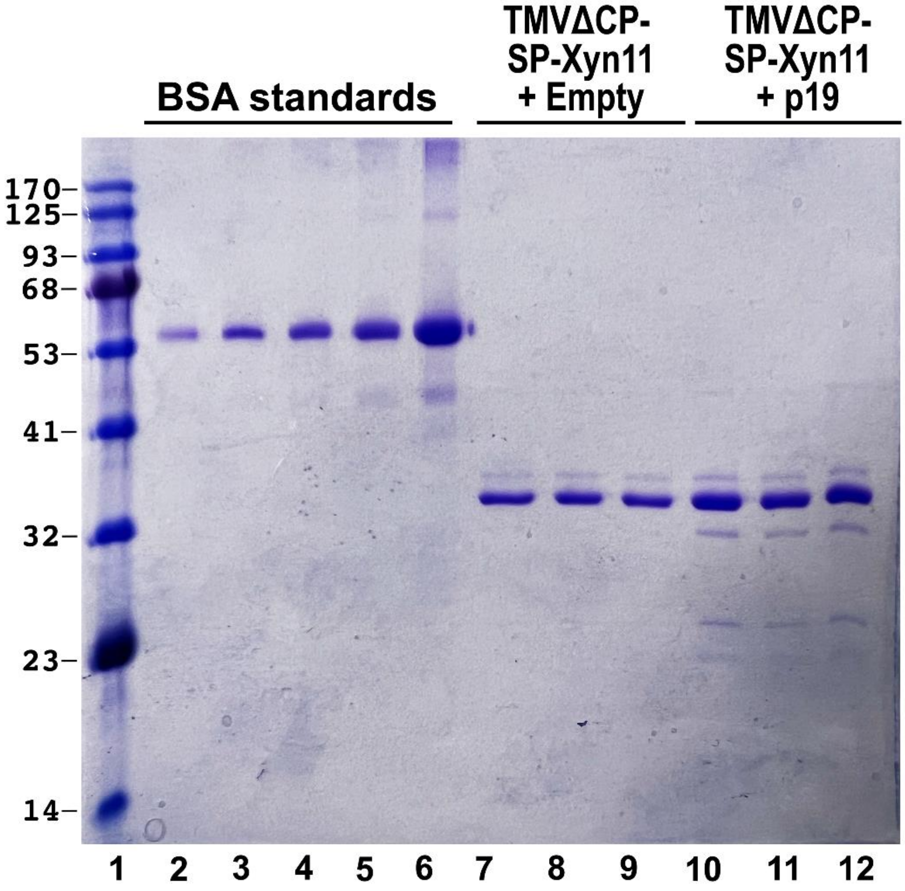

Fig. S4. Quantification of recombinant xylanase accumulating in *N. benthamiana* apoplast from plants co-agroinoculated with TEVΔCP-SP-Xyn11 and an empty plasmid or a plasmid to express TBSV p19 RNA silencing suppressor, as indicated. Leaves were harvested at 7 dpi, and protein extracts were prepared from the apoplastic fluid. Proteins were separated by SDS-PAGE, and the gel was stained with Coomassie brilliant blue. Lane 1, marker proteins with size in kDa on the left; lanes 2 to 6, BSA standards (0.24, 0.48, 0.72, 0.96 and 1.2 µg, respectively); lanes 7 to 12, biological replicates of apoplastic liquid (equivalent to 1.2 µl) harvested from plants co-agroinoculated with TMVΔCP-SP-Xyn11 and an empty plasmid (lanes 7 to 9) or TMVΔCP-SP-Xyn11 and a plasmid to express p19 (lanes 10 to 12).

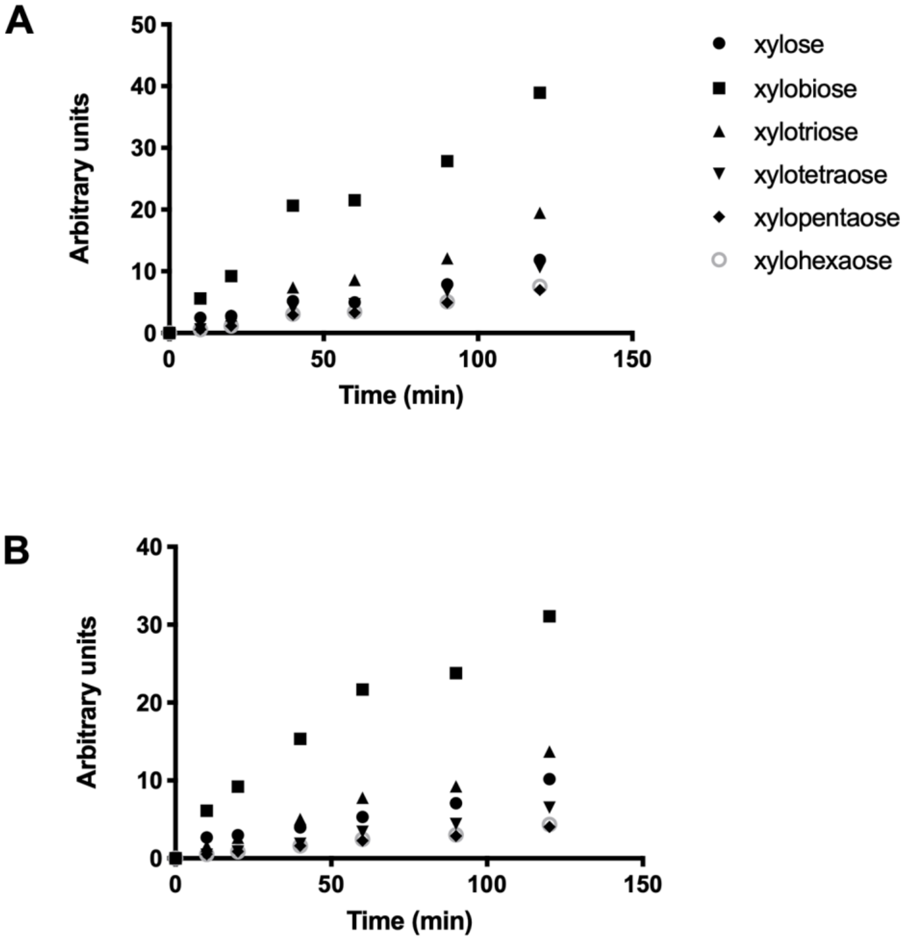

Fig. S5. Time-course production of XOS using (A) Xyn11_Nb and (B) Xyn11_Ec.

Table S1. Values of protein net charge (Z) were determined for Xyn11_Ec and Xyn11_Nb at different pH values.

pH	Xyn11_Ec	Xyn11_Nb
5.00	21.4	16.1
5.50	14.1	9.5
6.00	9.3	5.7
6.50	4.3	2.3
7.00	-0.2	-0.6
7.50	-3.0	-2.4
8.00	-4.6	-3.6
8.50	-6.3	-4.9
9.00	-9.3	-7.5
9.50	-15.5	-13.6
10.00	-26.2	-24.2
10.50	-37.0	-35.0

Table S2. PCR primers used for cloning purposes.

Primer	Orientation	Sequence	Product
D4375	Forward	5'-ctcagttcgtgttcttgtcaTGAGTTCTCAAATACCTTCTTTAAAAG-3'	Xyn11
D4376	Reverse	5'-ctacctcaagttgcaggaccTCATTTTGATACAGACTCTATG -3'	
D4377	Forward	5'-ctcagttcgtgttcttgtcaTGGCTTTGTGGTACTTGTTCAATAAAA-3'	SP-Xyn11
D4378	Reverse	5'-ctacctcaagttgcaggaccTCATTTTGATACAGACTCTATGACG-3'	
D4379	Forward	5'-ctcagttcgtgttcttgtcaTGGCTTTGTGGTACTTGTTCAATAAAAGGAG -3'	SP-Xyn11-AG
D4380	Reverse	5'-ctacctcaagttgcaggaccTCATGGGCTGGGAGAAGGGGAT-3'	
