# Supplementary figures and images for: Expression of an extremophilic xylanase in Nicotiana benthamiana and its use for the production of prebiotic xylooligosaccharides
Source: Sci Rep. 2022 Sep 21;12:15743. doi: 10.1038/s41598-022-19774-5 (PMC9492658; doi:10.1038/s41598-022-19774-5)

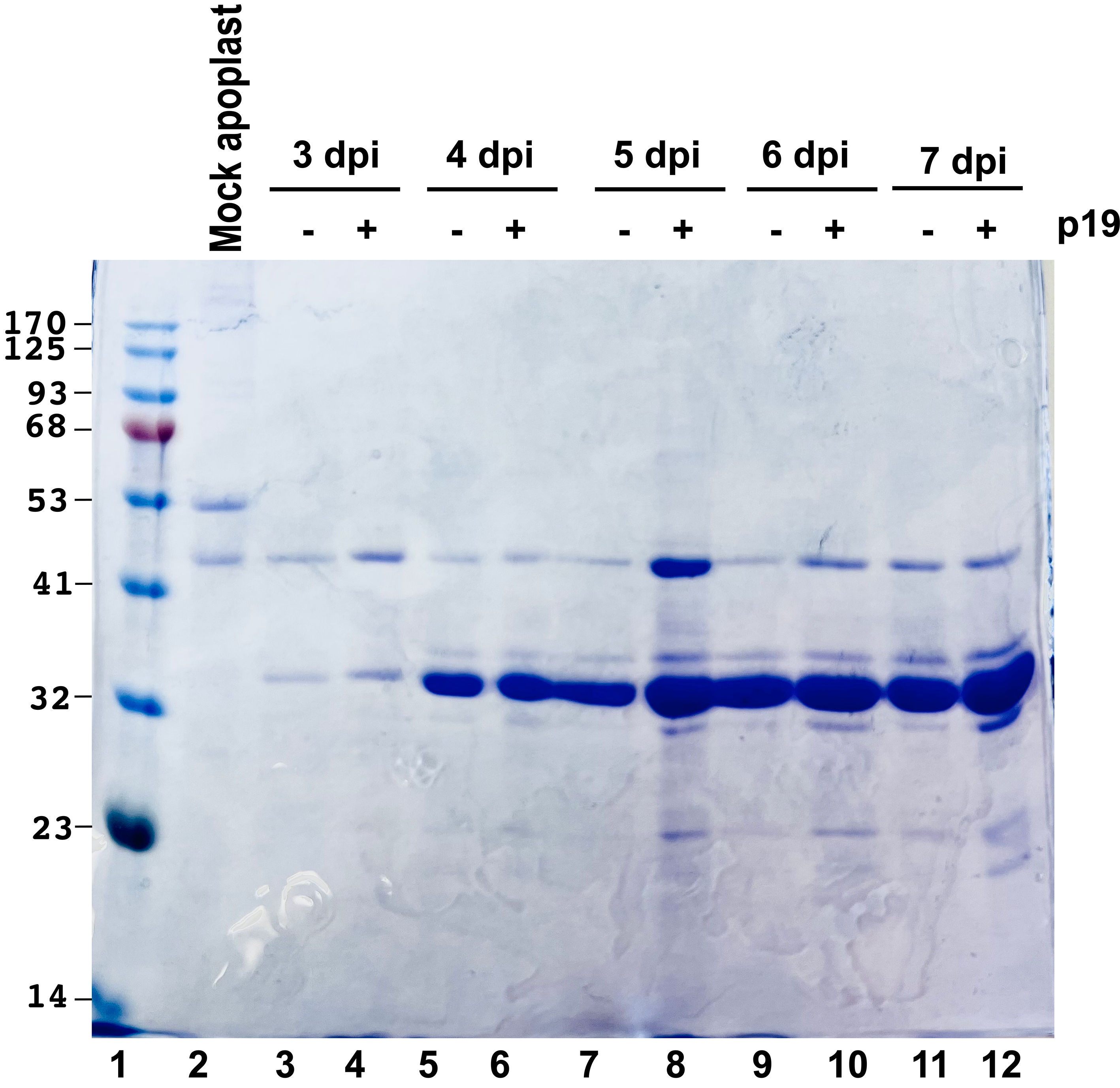

Supplement: Supplementary file 2 — Supplementary Information 2. [file 41598_2022_19774_MOESM2_ESM.tif]

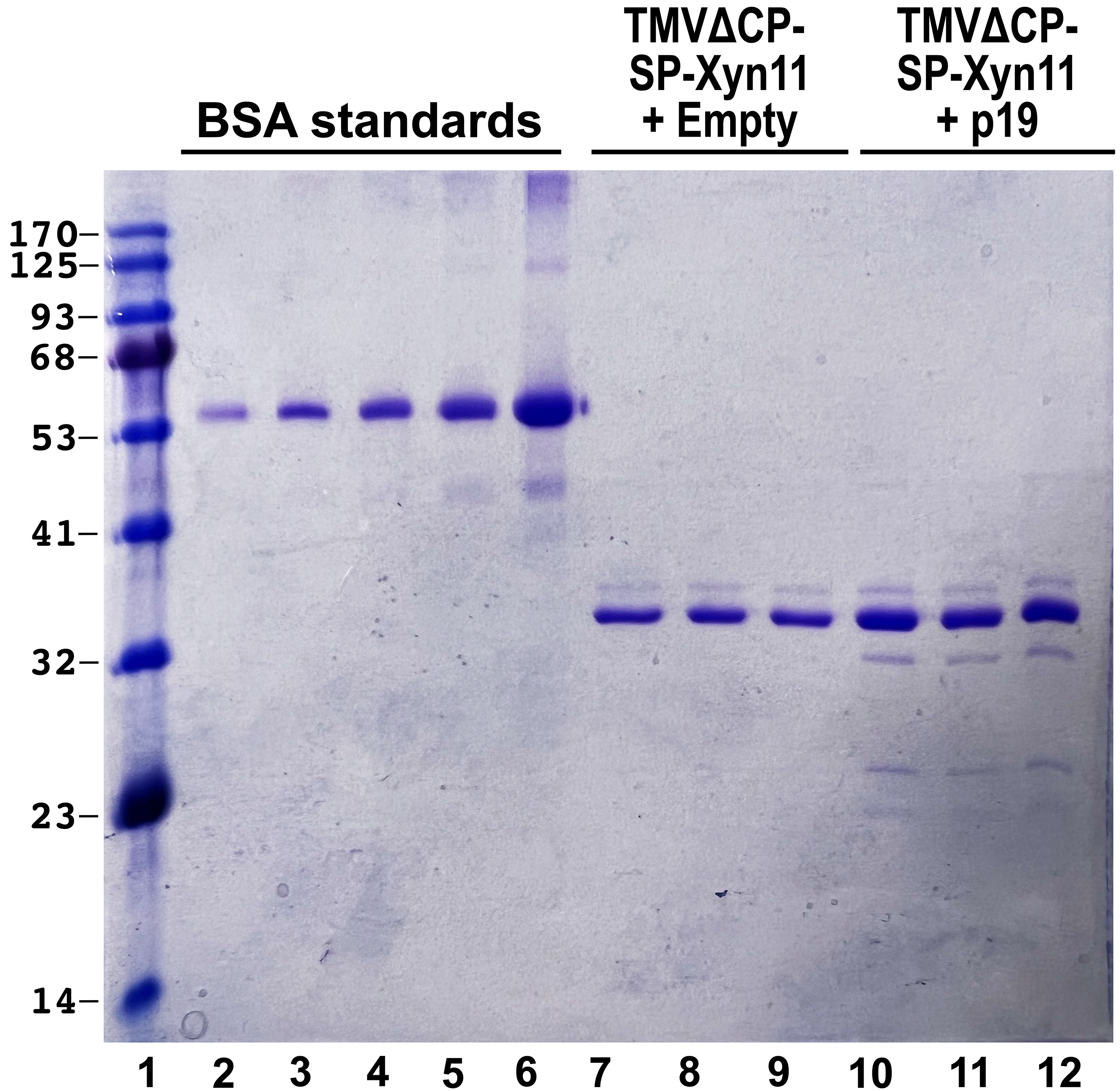

Supplement: Supplementary file 3 — Supplementary Information 3. [file 41598_2022_19774_MOESM3_ESM.tif]

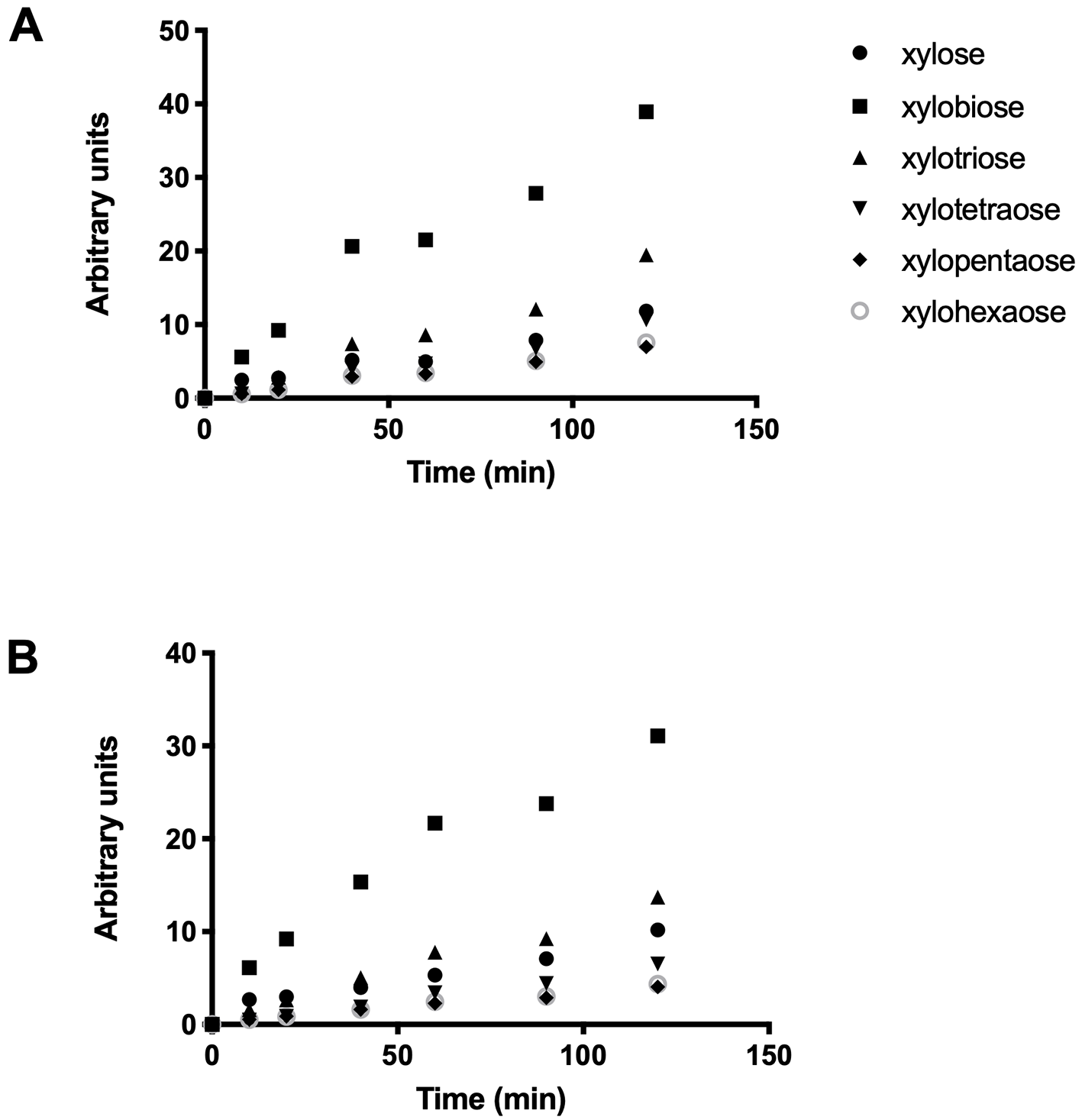

Supplement: Supplementary file 4 — Supplementary Information 4. [file 41598_2022_19774_MOESM4_ESM.tif]
